# The Mla system and its role in maintaining outer membrane barrier function in *Stenotrophomonas maltophilia*

**DOI:** 10.3389/fcimb.2024.1346565

**Published:** 2024-02-26

**Authors:** Xavier Coves, Uwe Mamat, Oscar Conchillo-Solé, Pol Huedo, Marc Bravo, Andromeda-Celeste Gómez, Ines Krohn, Wolfgang R. Streit, Ulrich E. Schaible, Isidre Gibert, Xavier Daura, Daniel Yero

**Affiliations:** ^1^ Institut de Biotecnologia i de Biomedicina (IBB), Universitat Autònoma de Barcelona (UAB), Cerdanyola del Vallès, Spain; ^2^ Departament de Genètica i de Microbiologia, Universitat Autònoma de Barcelona (UAB), Cerdanyola del Vallès, Spain; ^3^ Cellular Microbiology, Priority Research Area Infections, Research Center Borstel, Leibniz Lung Center, Leibniz Research Alliance INFECTIONS, Borstel, Germany; ^4^ Department of Microbiology and Biotechnology, University Institute of Plant Science and Microbiology, of Hamburg, Hamburg, Germany; ^5^ Catalan Institution for Research and Advanced Studies (ICREA), Barcelona, Spain; ^6^ Centro de Investigación Biomédica en Red de Bioingeniería, Biomateriales y Nanomedicina, Instituto de Salud Carlos III, Cerdanyola del Vallès, Spain

**Keywords:** *Stenotrophomonas maltophilia*, Mla system, biofilm, chelating agents, membrane permeability

## Abstract

*Stenotrophomonas maltophilia* are ubiquitous Gram-negative bacteria found in both natural and clinical environments. It is a remarkably adaptable species capable of thriving in various environments, thanks to the plasticity of its genome and a diverse array of genes that encode a wide range of functions. Among these functions, one notable trait is its remarkable ability to resist various antimicrobial agents, primarily through mechanisms that regulate the diffusion across cell membranes. We have investigated the Mla ABC transport system of *S. maltophilia*, which in other Gram-negative bacteria is known to transport phospholipids across the periplasm and is involved in maintaining outer membrane homeostasis. First, we structurally and functionally characterized the periplasmic substrate-binding protein MlaC, which determines the specificity of this system. The predicted structure of the *S. maltophilia* MlaC protein revealed a hydrophobic cavity of sufficient size to accommodate the phospholipids commonly found in this species. Moreover, recombinant MlaC produced heterologously demonstrated the ability to bind phospholipids. Gene knockout experiments in *S. maltophilia* K279a revealed that the Mla system is involved in baseline resistance to antimicrobial and antibiofilm agents, especially those with divalent-cation chelating activity. Co-culture experiments with *Pseudomonas aeruginosa* also showed a significant contribution of this system to the cooperation between both species in the formation of polymicrobial biofilms. As suggested for other Gram-negative pathogenic microorganisms, this system emerges as an appealing target for potential combined antimicrobial therapies.

## 1 Introduction

*Stenotrophomonas maltophilia* are Gram-negative bacteria widely distributed in the environment, characterized by an intrinsic, multidrug-resistant phenotype and substantial phylogenetic diversity (Gröschel et al., 2020). They are in general recognized as opportunistic human pathogens causing a wide range of infections (Brooke, 2021). In the clinical environment, these bacteria are often found colonizing the surface of medical devices, causing respiratory and urinary tract infections, especially in immunocompromised individuals or long-term hospital patients (Majumdar et al., 2022). Their virulence factors include extracellular enzymes, outer membrane (OM) proteins, outer membrane vesicles (OMV), lipopolysaccharides (LPS), fimbriae, flagella, adhesins, iron acquisition mechanisms, and the capacity to form biofilms (Trifonova and Strateva, 2019; Brooke, 2021). The best treatment for these infections is based on the combination trimethoprim/sulfamethoxazole, and more recently the use of minocycline, tigecycline, fluoroquinolones, and cefiderocol has also been recommended (Maraolo et al., 2023). Although they are considered low-virulence bacteria and therapeutic treatments are available, their intrinsic antimicrobial resistance and ability to form biofilms complicate the treatment of infections with *S. maltophilia*. In particular, the formation of highly heterogeneous biofilm on invasive devices, such as endotracheal tubes, catheters and central venous lines, increases its incidence as a nosocomial pathogen (Flores-Treviño et al., 2019; Alio et al., 2020; Brooke, 2021).

*S. maltophilia* is a model multidrug-resistant (MDR) organism with a variety of intrinsic resistance mechanisms (Gil-Gil et al., 2020). This MDR profile is an evolutionary consequence of the highly competitive environment in which these bacteria normally live, such as soil and especially the rhizosphere. Their intrinsic resistome includes, as in most MDR Gram-negative bacteria (Olivares et al., 2013), antibiotic inactivation genes, efflux pumps, resistance alleles or paralogs, and cell wall modification mechanisms. In fact, a reduced OM permeability is considered to be one of the major mechanisms of antimicrobial resistance in *S. maltophilia* (Sánchez, 2015; Brooke, 2021). Acquired and adaptive resistance to antibiotics also shapes the MDR phenotype of *S. maltophilia* strains, e.g., acquisition of new resistance genes by horizontal gene transfer, chromosomal mutations, transient regulation of gene expression, mechanisms of heteroresistance, OMV release, or biofilm formation (Devos et al., 2015; Sánchez, 2015; Martínez-Servat et al., 2018; Gil-Gil et al., 2020). The intrinsic resistance of *S. maltophilia* also extends to its interaction with other organisms. For example, *S. maltophilia* can cooperate in the formation of mixed biofilms with species that are highly competitive, such as *Pseudomonas aeruginosa*, *Staphylococcus aureus* and *Candida albicans* (McDaniel et al., 2020; Alio et al., 2023). Overall, the OM of *S. maltophilia* can be considered an important attribute of antimicrobial resistance and pathogenesis, and it also plays a role in the ecophysiology and adaptation to competitive environments. Therefore, the mechanisms that maintain the composition and integrity of the OM of *S. maltophilia* are key to survival and protection against external stressors.

Membrane phospholipid (PL) homeostasis in Gram-negative bacteria is fundamentally based on the enzymatic degradation or modification of glycerophospholipids and their transport between the inner and outer membranes (Powers and Trent, 2019; Lundstedt et al., 2021). These mechanisms guarantee not only the PL ratio between the two membranes, but also the asymmetry in the composition of PLs between the inner and outer leaflets of the OM. This asymmetry is essential to maintain the mechanical strength, fluidity and permeability of the OM (Nikaido, 2003). The OM of Gram-negative bacteria is their first line of defense, for example, to escape the effects of antimicrobials, detergents, and other harmful compounds (Powers and Trent, 2018). One of the known mechanisms of membrane homeostasis is the Maintenance of lipid asymmetry (Mla) system, which has been implicated in both retrograde and anterograde PL transport across the two membranes (Hughes et al., 2019; Tang et al., 2021). This system was originally discovered in *Escherichia coli* (Malinverni and Silhavy, 2009), and orthologous systems have been studied in various Gram-negative bacteria (Roier et al., 2016; Bernier et al., 2018; Baarda et al., 2019; Kamischke et al., 2019; Yero et al., 2021). It consists of the inner membrane ABC transporter MlaFEDB, the soluble periplasmic PL-binding component MlaC, and the OM protein MlaA/VacJ (Malinverni and Silhavy, 2009; Chong et al., 2015). Several studies have demonstrated that MlaC can indeed bind PL and have provided mechanistic information on how this protein transfers PL between other components of the Mla system (Ercan et al., 2019; Hughes et al., 2019; Yeow et al., 2023).

In this study, we investigated the homologous Mla system in *S. maltophilia* and its effect on resistance and virulence phenotypes in these bacteria. First, we characterized the periplasmic substrate-binding protein MlaC and its potential role in PL transport and membrane homeostasis in *S. maltophilia*. Based on mutational studies of the *mla* operon, we confirmed the contribution of the Mla system to the intrinsic resistance of *S. maltophilia* to certain membrane-damaging compounds and also to biofilm formation in the presence of sub-inhibitory concentrations of cation chelators. In addition, the role of this system in interspecific competition between *S. maltophilia* and *P. aeruginosa* during polymicrobial biofilm formation was investigated.

## 2 Materials and methods

### 2.1 Bacterial strains and growth conditions

Bacterial strains used in this study are listed in **Table 1**. *S. maltophilia* K279a (Abda et al., 2015) was used as a model strain to investigate the role of the *mla* operon in this species. *P. aeruginosa* PAO1 was used in competition and polymicrobial biofilm formation assays. Unless otherwise stated, all strains were routinely grown at 37°C in Miller’s LB medium (1% tryptone, 0.5% yeast extract, 1% NaCl) at 200 rpm on a rotatory shaker, or on LB supplemented with 1.5% (w/v) agar (LBA), except *E. coli* SM10 (λ*pir*)/pUX-BF13 (Bao et al., 1991) that was routinely cultured at 30°C. For biofilm formation assays, Brain Heart Infusion (BHI) broth from Oxoid (cat. No. CM1135) or modified BM2-glucose minimal medium (Overhage et al., 2007) (62 mM potassium phosphate buffer, pH 7.0, 2 mM MgSO_4_, 10 μM FeSO_4_, 0.4% glucose, supplemented with 0.5% casamino acids) was used. In general, bacterial growth was measured in a Novaspec II spectrophotometer at 600 nm (OD_600nm_). When necessary, antibiotics were added to the agar plates at the indicated concentration.

**Table 1:** Bacterial strains and plasmids used in this work.

Strain or plasmid	Genotype and/or relevant characteristics	Reference or source
Strains		
*Stenotrophomonas maltophilia*		
K279a	Wild-type. Clinical isolate and the genetic reference strain	Laboratory collection and (Abda et al., 2015)
Δ*mlaF-B*	K279aΔ*mlaF-B* (mutant for *mlaF-B* genes)	This study
Δ*mlaA*	K279aΔ*mlaA* (mutant for the *mlaA*/*vacJ* gene)	This study
K279a::sfGFP	sfGFP-tagged strain K279a	(Mamat et al., 2023)
Δ*mlaF-B*::sfGFP	sfGFP-tagged strain K279a Δ*mlaF-B*	This study
*Xanthomonas campestris pv. campestris*		
8523 pL6engGUS	*rpfF* mutant, plasmid pLAFR6 carrying *engXCA*:*gusA* fusion. DSF-reporter strain	(Slater et al., 2000)
*Escherichia coli*		
DH5α	F^-^ Φ80*lacZ*ΔM15 Δ(*lacZYA-argF*) U169 *recA1 endA1 hsdR17*(r_K_^-^ m_K_^+^) *phoA supE44 thi-1 gyrA96 relA1*λ^-^	Laboratory collection and (Hanahan, 1983)
SY327	Δ(*lac pro*) *argE*(*Am*) *recA56 rifR nalA* λ *pir*	(Miller and Mekalanos, 1988)
BL21(DE3) pLysS	F^–^, *ompT*, *hsdSB* (rB^–^, mB^–^), *dcm*, *gal*, λ(DE3), pLysS, Cm^r^	Novagen
*Pseudomonas aeruginosa*		
PAO1	Wild-type and genetic reference strain	(Yero et al., 2021)
PAO1:: tdTomato	tdTomato-tagged strain PAO1	This study
Plasmids		
pGPI-SceI-XCm	Mobilizable suicide vector; carries the R6Kγ origin of replication, the I-SceI recognition site and a *xylE* reporter gene, Cm^r^, Tp^r^	(Hamad et al., 2010)
pΔ*mlaF-B*-US’DS’	pGPI-SceI-XCm containing the upstream and downstream flanking DNA regions of the genes *mlaF-B* (*smlt4670-4674*)	This study
pΔ*mlaA*-US’DS’	pGPI-SceI-XCm containing the upstream and downstream flanking DNA regions of the *mlaA* (*smlt4675*) gene	This study
pRK2013	RK2-derived helper plasmid carrying the *tra* and *mob* genes for mobilization of plasmids containing *oriT*, Kan^r^	(Figurski and Helinski, 1979)
pUX-BF13	R6Kγ-based helper plasmid containing the Tn*7* transposase genes tnsABCDE for transposition of mini-Tn*7* elements, Amp^r^	(Bao et al., 1991)
pDAI-SceI-SacB	Mobilizable broad-host range plasmid; carries the gene for the I-SceI homing endonuclease and the *sacB* gene, Tet^r^	(Flannagan et al., 2008; Hamad et al., 2010)
pBBR1MCS1	Broad-host range cloning vector used for complementation, low copy, Cm^r^	(Kovach et al., 1994)
pBBR1MCS1-*mlaF-B*	pBBR1MCS-1 with genes *smlt4670-4674* inserted between sites *Xba*I and *HindIII*, Cm^r^	This study
pBAD18-Cm	Expression vector containing the arabinose pBAD promoter and *araC*, Cm^r^	(Guzman et al., 1995)
pBBR1-BAD-Cm	pBBR1MCS1 with the pBAD18Cm cassette	This study
pBBR1-BAD-Cm-*mlaA*	pBBR1-pBAD-Cm with the *mlaA* (*smlt4675*) CDS inserted between sites *Nhe*I and *Hind*III, Cm^r^	This study
pET28b	Bacterial expression vector with T*7*-lacO promoter, hexa-His-tag (Nterm and Cterm) with Thrombin cleavage site (N-terminal on backbone), Kan^r^	Novagen
pET28b-H6-MlaC	Plasmid for MlaC (Smlt4673) protein production in *E. coli*	This study
pUC18T-mini-Tn7T-Gm-rpoD-sfGFP	Mini-Tn*7* delivery plasmid containing the codon-optimized gene for sfGFP, Amp^r^, Gm^r^	(Mamat et al., 2023)
pUC18T-mini-Tn7T-Gm-Pc-tdTomato	Mini-Tn*7* delivery plasmids containing the codon-optimized tdTomato gene, Amp^r^, Gm^r^	(Mamat et al., 2023)

### 2.2 Construction of markerless deletion mutants and complementation

Markerless *S. maltophilia* K279a mutants were constructed using the pGPI-SceI/pDAI-SceI-SacB system, which was initially described for species of the genus *Burkholderia* (Flannagan et al., 2008; Aubert et al., 2014). Briefly, pΔ*mlaF-B*-US’DS’ and pΔ*mlaA-*US’DS’ deletion plasmids were derived from the mobilizable suicide vector pGPI-SceI-XCm containing upstream and downstream flanking regions of the target genes in the K279a genome and maintained in *E. coli* SY327 (see **Tables 1**, **2** for plasmid construction and primer details, respectively). The deletion plasmids were then transferred to the recipient *S. maltophilia* K279a by triparental mating using *E. coli* DH5α/pRK2013 as a helper strain and *E. coli* SY327 carrying the deletion plasmid as a donor strain. The K279a co-integrates were selected at 37°C on LBA plates containing 5 µg/mL norfloxacin in order to counterselect against *E. coli* donor and helper strains and 60 µg/mL chloramphenicol to select for K279a transconjugants. To confirm the integration of the suicide plasmids, single colonies were streaked and sprayed with 0.45 M pyrocatechol. Next, the plasmid pDAI-SceI-SacB was introduced into *S. maltophilia* K279a co-integrates by triparental mating using *E. coli* DH5α/pDAI-SceI-SacB and *E. coli* DH5α/pRK2013 as donor and helper strains, respectively. Transconjugants were selected at 30°C on LBA plates containing 5 µg/mL norfloxacin and 50 µg/mL tetracycline. The loss of the integrated pGPI-SceI-XCm plasmid was confirmed by the absence of yellow color upon pyrocatechol exposure and susceptibility to 60 µg/mL chloramphenicol. The presence of the mutant allele was confirmed by PCR screening and DNA sequencing. Markerless mutants were obtained by curing the plasmid pDAI-SceI-SacB using sucrose counterselection.

*In trans* complementation of the deleted genes was achieved by cloning the entire region containing the wild-type *mlaF-B* operon and the *mla*A gene into the broad-host range plasmids pBBR1MCS1 and pBBR1-BAD-Cm, respectively. To generate the expression vector pBBR1-BAD-Cm, in which *mlaA* transcription is driven by the arabinose promoter, the pBAD promoter and AraC-encoding cassette from pBAD18-Cm were cloned into the vector pBBR1MCS1 on compatible *Spe*I/*Xho*I sites using standard techniques and primers pBAD18-Up and pBAD18-Lw (**Table 2**).

**Table 2:** Primers used in this study.

Primer	Sequence^a^ (5’-3’)	Application
US’-mlaFEDCB-U	CCAgcggccgcACTTTTCAAATAC	Upstream flanking region, forward primer to create pΔ*mlaF-B*-US’DS’, *Not*I
US’-mlaFEDCB-L	GGGggtaccTAACGTTTTCTAGACATCGA	Upstream flanking region, reverse primer to create pΔ*mlaF-B*-US’DS’, *Kpn*I
DS’-mlaFEDCB-U	CGAggtaccATGAACGTAGTGCGCAC	Downstream flanking region, forward primer to create pΔ*mlaF-B*-US’DS’, *Kpn*I
DS’-mlaFEDCB-L	GCAtctagaGTCTGGCCGAAGTCCTCAT	Downstream flanking region, reverse primer to create pΔ*mlaF-B*-US’DS’, *XbaI*
US’-mlaA-U	ATTgcggccgcTCGACCCTGCTCAACATCCAG	Upstream flanking region, forward primer to create pΔ*mlaA*-US’DS’, *Not*I
US’-mlaA-L	GCTggtaccGGAGAGTGCGCACGACGTTCA	Upstream flanking region, reverse primer to create pΔ*mlaA*-US’DS’, *Kpn*I
DS’-mlaA-U	CTGggtaccTACAGCGACGCATGAAAAACC	Downstream flanking region, forward primer to create pΔ*mlaA*-US’DS’, *Kpn*I
DS’-mlaA-L	GAGtctagaCGTGAGCACTTCGATCCAGGA	Downstream flanking region, reverse primer to create pΔ*mlaA*-US’DS’, *XbaI*
Ext-mlaFEDCB-U	CGCAACCATGAAGATGAAACT	Forward primer outside the deleted region for mutant verification
Ext-mlaFEDCB-L	CTCAGTTCGTTGTAGCCAGCC	Reverse primer outside the deleted region for mutant verification
Ext-mlaA-U	AATGGCAAGTAACGCACTGGC	Forward primer outside the deleted region for mutant verification
Ext-mlaA-L	GCTATGCTGGTGGGCCATTC	Reverse primer outside the deleted region for mutant verification
mlaFEDCB_Comp_U	AATTtctagaCCAGTGTCGCGTGCAGCCAGC	Forward primer for cloning genes *smlt4670-4674* into pBBR1MCS1 including its own promoter, *XbaI*
mlaFEDCB_Comp_L	GGGaagcttGCGCACGACGTTCATGTCA	Reverse primer for cloning genes *smlt4670-4674* into pBBR1MCS1, *HindIII*
mlaA_Comp_U	TGGtctagaTGGATCTGGCTGAGGTCGAAC	Forward primer for cloning gene *smlt4675* into pBBR1-BAD-Cm including its own promoter, *XbaI*
mlaA_Comp_L	TGCaagcttGAAAGGACTCAGTGCGTC	Reverse primer for cloning gene *smlt4675* into pBBR1-BAD-Cm, *HindIII*
pBAD18-Up^b^	CCCactagtATGTCGGCGATATAG	Forward primer for cloning pBAD promoter and *araC* from pBAD18-Cm into pBBR1MCS1, *SpeI*
pBAD18-Lw^b^	ATGctcgagGGAAATGTTGAATAC	Reverse primer for cloning pBAD promoter and *araC* from pBAD18-Cm into pBBR1MCS1, *XhoI*
MlaC_His_Up	CGGtcatgaGCCATCATCATCATCATCATGCCGCCGCCCCCGCCGCT	Forward primer for cloning *mlaC* into pET28b including 6 codons for His, *BspHI*
MlaC_ Lw	GAGctcgagTTACTTGCCATTGCCCGCGGGCCCGGCCTGCAT	Reverse primer for cloning *mlaC* into pET28b, *XhoI*
PAO1-PA5548-Ctr	TTACCTGCGACGTTATCTGAAGC	Forward primer for amplification of the left flanking mini-Tn*7* region
PAO1-glmS-Ctrl1	GCTGAAGCTCAAGGAAATTTCC	Reverse primer for amplification of the right flanking mini-Tn*7* region

a Restriction site is shown in lower case letters.

b The primers have been described previously (Yero et al., 2021).

### 2.3 Construction of fluorescently labelled strains

The fluorescently labelled *S. maltophilia* wild-type strain K279a::sfGFP was constructed previously with a mini-Tn*7* delivery system that has been optimized for work with bacteria of the family *Xanthomonadaceae* (Mamat et al., 2023). The same procedure using transfer of the mini-Tn*7* delivery plasmid pUC18T-mini-Tn*7*T-Gm-Pc-sfGFP to *S. maltophilia* K279a Δ*mlaF-B* by four-parental mating was applied herein to label the *mla* mutant strain with sfGFP. For the construction of *P. aeruginosa* PAO1::tdTomato, the plasmid pUC18T-mini-Tn*7*T-Gm-Pc-tdTomato was transferred to the *P. aeruginosa* PAO1 wild-type strain by conjugation, involving *E. coli* DH5α/pRK2013 (Figurski and Helinski, 1979) and *E. coli* SM10 λ*pir*/pUX-BF13 as helper strains, *E. coli* DH5α/pUC18T-mini-Tn*7*T-Gm-Pc-tdTomato (Mamat et al., 2023) as the donor, and *P. aeruginosa* PAO1 as the recipient strain. The strains were grown overnight in LB media with kanamycin (30 µg/mL) for *E. coli* DH5α/pRK2013, 100 µg/mL of ampicillin for *E. coli* SM10 λ*pir*/pUX-BF13, and ampicillin (100 µg/mL) and gentamycin (15 µg/mL) for *E. coli* DH5α/pUC18T-mini-Tn*7*T-Gm-Pc-tdTomato. The cells from 1 mL of each *E. coli* culture and 330 µL of *P. aeruginosa* PAO1 were harvested by centrifugation. For preparation of the mating mixture, the cell pellets of all strains were pooled in 1 mL of LB medium, sedimented by centrifugation, resuspended in 100 µL of super optimal broth (SOB) (0.5% yeast extract, 2% tryptone, 10 mM NaCl, 2.5 mM KCl, 20 mM MgSO_4_) and then spotted onto an SOB agar plate essentially as described elsewhere (Aubert et al., 2014). The *P. aeruginosa* PAO1 transconjugants were selected at 37°C on Vogel-Bonner minimal medium (VBMM) (3 g/L trisodium citrate, 2 g/L citric acid, 10 g/L K_2_HPO_4_, 3.5 g/L NaNH_4_PO_4_·4H_2_O, 1 mM MgSO_4_, 100 µM CaCl_2_, pH 7.0) agar plates containing 30 µg/mL of gentamycin (Choi and Schweizer, 2006). Chromosomal insertion of the mini-Tn*7* transposon downstream of the *glmS* gene for glutamine–fructose-6-phosphate aminotransferase was verified by PCR, using the primer pairs PTn7R (Choi and Schweizer, 2006) and PAO1-glmS-Ctrl1, and PTn7L (Choi and Schweizer, 2006) and PAO1-PA5548-Ctr to amplify the flanking mini-Tn*7* regions (**Table 2**), respectively.

### 2.4 Growth curves and competition assays

To compare the growth of mutant strains with that of their parent strains, fresh overnight cultures were adjusted to an OD_600nm_ of 0.01 with appropriate media, followed by transfer of 200 μL of each diluted culture to each well of a conventional 96-well microtiter plate (triplicate samples). The plates were incubated for 24 hours in a Multiskan FC microplate photometer (Thermo Fisher Scientific) with constant circular shaking at 30°C or 37°C for bacterial growth, and the OD_550nm_ was measured every 15 min.

For growth competition assays on agar plates, overnight cultures of *S. maltophilia* were adjusted to OD_600nm_ of 0.01 and spread onto LBA using a sterile cotton swab to prepare a bacterial lawn. Five microliters of logarithmic cultures of *P. aeruginosa* PAO1 were spotted onto the competitor lawns using a micropipette. Plates were incubated for 24 hours at 37°C. For growth competition assays in liquid cultures, overnight cultures in 0.5 × BHI medium were diluted to an OD_600nm_ of 0.05 (∼5 × 10^8^ CFU/mL) and were inoculated simultaneously at an equal ratio (1:1 v/v). Samples were collected at 24 hours, serially diluted in PBS, and then seeded onto LBA with 60 µg/mL chloramphenicol for *P. aeruginosa* PAO1 and LBA with 50 µg/mL streptomycin for *S. maltophilia* K279a. The agar plates were incubated at 37°C for 24 hours and the number of viable cells was determined.

### 2.5 Plating efficiency assay

For plating efficiency tests, strains were grown overnight in LB medium at 37°C, serially diluted 10-fold, and then replica plated on LBA or MacConkey agar (MacConkey broth from Oxoid with 1.5% (w/v) agar). Plates were then incubated overnight at 37°C.

### 2.6 Antimicrobial susceptibility testing

The minimum inhibitory concentration (MIC) of a broad spectrum of antibiotics was determined using the broth microdilution method in accordance with CLSI guidelines (CLSI, 2015) and EUCAST recommendations for colistin (The European Committee on Antimicrobial Susceptibility Testing and Clinical and Laboratory Standards Institute, 2016). Briefly, MICs were determined in sterile 96-well plates by two-fold serial dilutions of the corresponding antibiotic in cation adjusted Mueller-Hinton broth (CAMHB). To prepare CAMHB, Mueller-Hinton Broth (MHB) from Oxoid (cat. No. CM0405) was supplemented with calcium and magnesium to final concentrations of 25 and 12.5 mg/L, respectively. First, two-fold serial dilutions of antibiotics were prepared in 100 μl of CAMHB in microtiter plate wells. Overnight cultures in CAMHB were diluted in the same media to contain an initial inoculum of 5 × 10^5^ cells/mL. Each well containing the corresponding two-fold serial antibiotic dilution was filled with 100 µL of the cell suspension (final volume of 200 µL/well). MIC plates were read after 20 hours of incubation at 37°C without shaking. The MIC was determined to be the lowest concentration at which no bacterial growth occurred, as assessed by visual inspection and confirmed by adding 30 µL of 0.01% resazurin to each well to determine cell viability. Susceptibility to the membrane-damaging agents EDTA (ethylenediaminetetraacetic acid) and SDS (sodium dodecyl sulfate) was also determined by the broth microdilution method using MHB.

### 2.7 NPN outer membrane permeability assay

Uptake of 1-*N*-phenylnaphthylamine (NPN) was investigated as previously described (Helander and Mattila-Sandholm, 2000), with a few modifications (Loh et al., 1984; Fernández et al., 2012). Stock solutions of NPN at 50 mM and the uncoupler carbonyl cyanide *m*-chlorophenylhydrazone (CCCP) at 5 mM, both reagents purchased from Sigma-Aldrich, were prepared in advance in acetone. NPN was then diluted in 5 mM HEPES buffer (pH 7.2) to a concentration of 40 µM to prepare a working solution. Bacterial overnight cultures were diluted 100-fold in LB medium with appropriate antibiotic and grown to mid-exponential phase (OD_600nm_ of 0.6). Cells were harvested by centrifugation at room temperature and resuspended in 5 mM HEPES buffer (pH 7.2) supplemented with CCCP at 5 µM (HEPES-CCCP buffer). The cell suspension (OD_600nm_ = 0.5) was left at 23°C for an hour before adding any reagent. The cell suspension (100 µL), 40 µM NPN working solution (50 µL) and HEPES-CCCP buffer (50 µL) were mixed in flat-bottomed black 96-well plates immediately before measuring the fluorescence (excitation, 340 nm; emission, 415 nm) in a Victor 3V 1420 multilabel plate reader (PerkinElmer^®^). To increase membrane permeability, 0.5 mM EDTA was added. Values were recorded up to 3 min to calculate NPN uptake kinetics. The results were expressed as relative fluorescence units (fluorescence value for the cell suspension with NPN, minus the corresponding value for the control), per triplicate samples, and two-way analysis of variance (ANOVA) with Bonferroni’s multiple-comparison test was applied to the data (GraphPad Prism v 9.0; GraphPad Inc, San Diego, USA).

### 2.8 Bioassay for diffusible signal factor detection

DSF determination was performed using the reporter strain *Xanthomonas campestris pv. campestris* (*Xcc*) 8523 pL6engGUS as previously described (Huedo et al., 2015). The Xcc reporter strain was routinely grown in NYG medium (0.5% peptone, 0.3% yeast extract and 2% glycerol) supplemented with 10 μg/mL of tetracycline at 28°C. Strains to be tested were pin inoculated onto NYG agar plates containing 80 μg/mL of 5-bromo-4-chloro-3-indolyl-β-d-glucopyranoside (X-Glu) and the DSF-reporter strain and incubated for 24 hours at 28°C. The presence of a blue halo around the colony indicated DSF activity.

### 2.9 Congo red agar plate assay

Overnight cultures were streaked onto BHI agar containing 3% sucrose and 0.08% (wt/v) Congo red dye. Plates were incubated at 30°C for 24 hours, and colonies were then examined for absorption of Congo red dye.

### 2.10 Biofilm formation assays

To evaluate single-species biofilm formation under static conditions, overnight cultures of the strains were grown aerobically (200 rpm) in 0.5 × BHI broth or modified BM2-glucose minimal medium at 37°C, followed by dilution of each culture with the corresponding fresh medium to an OD_600nm_ of 0.05. Only the overnight cultures of the complemented strain carrying the plasmid pBBR1MCS1-*mlaF-B* were supplemented with 60 µg/mL of chloramphenicol. Sterile, untreated 96-well flat-bottom microtiter plates were then inoculated with the fresh bacterial suspensions (200 μL per well) and incubated at 30°C for 24 hours in a humidified atmosphere. Quantification of the biofilm biomass was performed by crystal violet (CV) staining as described previously (Yero et al., 2020). The amount of biofilm was quantified by measuring the OD_550_ of dissolved CV using a microplate reader (Multiskan FC microplate photometer, Thermo Fisher Scientific).

Mixed biofilms of *S. maltophilia* and *P. aeruginosa* strains were investigated with sfGFP- or tdTomato-labelled cells, respectively, in ibiTreat µ-Slide 8 well plates (Ibidi, Cat.No: 80826). To grow mixed biofilms of fluorescently tagged *S. maltophilia* and *P. aeruginosa* strains, preparation of the samples included overnight pre-cultures of the strains grown with agitation at 37°C in 0.5 × BHI medium and containing 60 µg/mL of chloramphenicol for the complemented mutant strain. The next day, the OD_600nm_ was adjusted to 0.05 for the initial inocula in 0.5 × BHI medium at 30°C, and 200 µL of each strain (in duplicate) were inoculated into 8-well chambers and incubated at 30°C in a humidified atmosphere. Every 12 hours, the medium was aspirated from each chamber and fresh medium was added, up to a total incubation time of 72 hours. Prior to observation under the microscope, all excess medium was aspirated to image the biofilm. Imaging was performed with a TCS SP5 inverse confocal laser-scanning microscope (Leica Microsystems, Mannheim, Germany) equipped with a Leica 63x/NA 1.40 HCX Plan Apochromat CS oil immersion objective. The sfGFP protein was excited with 488 nm laser light, and fluorescence emission was detected between 495 and 550 nm. The excitation wavelength of laser light was 561 nm for tdTomato and emission of its fluorescence was detected between 580 and 630 nm. The fluorescent signal from a minimum of three fields per chamber was collected (50 z-stacks) for processing. The images were analyzed with LAS AF software (version 2.73) and three-dimensional biofilm images were generated from confocal image stacks using the *daime* computer program (Daims et al., 2006). Images were split into individual color channels for biovolume quantification using the *daime* software. Image acquisition and processing was carried out at the core facility Fluorescence Cytometry of the Research Center Borstel (Borstel, Germany). ANOVA with Bonferroni’s multiple-comparison test was applied to the data from all biofilm experiments using GraphPad Prism v 9.0.

### 2.11 Electron microscopy of bacterial cells

The visualization of *S. maltophilia* cells was performed on a Scanning Electron Microscope (SEM) LEO SEM 1525 (Zeiss, Germany) at 5 kV at the Hamburg Zoological Institute (Hamburg, Germany). Overnight cultures of each strain were prepared in 5 mL of LB medium with or without a sub-inhibitory concentration of 0.5 mM EDTA. The suspensions were centrifuged at 13,000 rpm for 2 min, and the cell sediments were resuspended in 500 µL of sterile PBS. Samples were fixed in 1% paraformaldehyde and gradually dehydrated in increasing concentrations of ethanol. The dehydration step was completed by drying the pellets at the critical point with a Balzers CPD 030 Critical Point Dryer instrument. Prior to visualization, the samples were coated with gold particles using an SCD 050 Sputter Coater (Bal-Tec, United States).

Visualization of internal structures of the different strains of *S. maltophilia* was carried out using the transmission electron microscope (TEM) Biotwin CM120 (Philips, Netherlands) operating at 120 kV in the facilities of the Biocenter Klein Flottbek of the University of Hamburg (Germany). Overnight cultures were prepared under the same conditions as for SEM. Cells were fixed overnight with 2.5% glutaraldehyde and post-fixed with 1% osmium tetraoxide for 2 hours. Prior to observation, the cells were dehydrated in ethanol gradients. Ultrathin sections were obtained using a UC7 ultramicrotome (Leica Microsystems) and deposited on a nickel-coated grid and counterstained with uranyl acetate and lead citrate. The images obtained with the Gatan MSC 794 camera were recovered with the built-in Digital Micrograph software.

### 2.12 Recombinant MlaC expression and purification

To produce N-terminally His-tagged MlaC (Smlt4573) protein in *E. coli* BL21(DE3)pLysS, the coding region was cloned into the *Nco*I/*Xho*I sites of pET28b using standard techniques and primers MlaC_His_Up and MlaC_Lw (**Table 2**). Since MlaC is naturally located in the periplasm, the signal sequence was removed from the coding sequence of the recombinant protein. The cells of an overnight culture of *E. coli* BL21(DE3)pLysS containing the pET28b-H6-MlaC plasmid were diluted 1:100 in 2 L of pre-warmed LB and grown to mid-exponential phase (OD_600nm_ = 0.6). Isopropyl-ß-d-thiogalactopyranoside (IPTG) was added to the culture at a final concentration of 1 mM for protein expression induction. After four hours, cells were harvested (OD_600nm_ = 3.5) and washed twice with sterile PBS. In order to verify the presence of the MlaC protein in the soluble fraction, the cell pellet was resuspended in lysis buffer (5 mM imidazole, 0.5 M NaCl, 0.1% Triton X-100, 5% glycerol, 20 mM Tris-HCl, pH 7.9), lysed by sonication in an ice bath, and separated by 12% SDS-PAGE. Protein purification was performed by the Protein Production Platform (PPP) of Nanbiosis facilities at the Institute of Biotechnology and Biomedicine of the Universitat Autònoma de Barcelona (UAB, Spain). Briefly, cells were disrupted by sonication in lysis buffer containing 4 µg/mL lysozyme, 8 µg/mL DNase and 2 mM MgCl_2_. Protein was purified from the soluble fraction by immobilized metal affinity chromatography (IMAC) on a HisTrap HP 5 mL (GE, 17-5248-01) column with washing buffer (40 mM imidazole, 0.33 M NaCl, 13.3 mM Tris-HCl, pH 7.9) and elution buffer (125 mM imidazole, 62.5 mM NaCl, 2.5 mM Tris-HCl, pH 7.9). Eluted protein was immediately dialyzed against 50 mM Na_2_HPO_4_ (pH 7.0) to remove the imidazole.

### 2.13 Delipidation of purified MlaC protein

Recombinant MlaC protein produced in *E. coli* was delipidated using an HPLC system and a C18 column (Phenomenex Jupiter 5U C18 300A) in 0.1% TFA. Protein was eluted from the column with a gradient of acetonitrile in 0.1% TFA.

### 2.14 Mass spectrometry and *E. coli* phospholipid identification

Protein identity was confirmed by peptide mass fingerprinting using matrix-assisted laser desorption ionization–time of flight mass spectrometry (MALDI-TOF MS) at the Proteomics Laboratory of the Consejo Superior de Investigaciones Científicas and UAB. Analysis of MlaC-bound PLs was done under denaturing conditions by MALDI-TOF MS in the negative ion mode with 9-aminoacridine as matrix. The identification of *E. coli* PLs present in the sample was done according to Oursel et al. (2007) and Gidden et al. (2009), using Lipidomics Gateway (http://www.lipidmaps.org) based on the m/z values of MS spectra.

### 2.15 Biophysical characterization of phospholipid binding to MlaC protein

The binding of phosphatidylethanolamines PE14/14 and PE16/16 to MlaC was first evaluated by nano Differential Scanning Fluorimetry (nanoDSF), which determines the melting temperature of the protein in the presence and absence of compound. The reaction buffer was optimized to 50 mM MOPS pH 7.0, 100 mM NaCl and 0.05% Tween, for a final reaction volume of 10 μl; final protein and compound concentrations were 5 μM and 100 μM, respectively, and DMSO concentration was 1%; the reaction temperature was 20-95°C, with a heating rate of 1°C min^-1^. Binding and corresponding K_d_ values were then evaluated by MicroScale Thermophoresis (MST), which measures protein movement along a temperature gradient in the presence and absence of compound. The reaction buffer was as for nanoDSF; final dye and protein concentrations were 5 nM and 100 nM, respectively; MST excitation power was 20%. Both assays were performed by Proteros Biostructures GmbH (Germany).

### 2.16 Bioinformatics analysis

*S. maltophilia* K279a homologues of *P. aeruginosa* PAO1 genes coding for Ttg2ABCDE (PA4452-PA4456) or VacJ (PA2800) and *E. coli* K-12 substr. MG1655 genes encoding MlaFEDCB (b3195- b3191) or MlaA (b2346) were identified as follows. Protein sequences were first analyzed using BLAST, PSI-BLAST and CDD within NCBI (http://ncbi.nlm.nih.gov/). Orthologous sequences in other species were searched using BLASTp and initially aligned with Clustal Omega (https://www.ebi.ac.uk/Tools/msa/clustalo/). Promoter prediction was done by means of PRODORIC database (Dudek and Jahn, 2022). Operon organization was inferred from the BioCyc data collection (Caspi et al., 2016) and MicrobesOnline database (Alm et al., 2005). Transcription units were identified by mapping reads from previously published RNA-seq experiment (GEO GSE206442) (Coves et al., 2023) on the K279a chromosome and visualized by the Artemis genome browser (Rutherford et al., 2000).

Protein structures of MlaC orthologues from *P. aeruginosa* (6HSY) (Yero et al., 2021), *E. coli* (5UWA) (Ekiert et al., 2017) and *Ralstonia solanacearum* (2QGU) were obtained from the RCSB PDB database (https://www.rcsb.org/). For structural comparisons, all selected structures are lipid-bound and hydrogen atoms were removed from the 6HSY structure. Multiple sequence alignment was constructed using hmmalign tool from the HMMER v3.3.2 package (Mistry et al., 2013) using Pfam PF05494.15 profile (https://www.ebi.ac.uk/interpro/) and presented with Jalview (Waterhouse et al., 2009). DeepMind’s AlphaFold (Jumper et al., 2021) version 2.2.4, locally installed and running in a Docker container, was used to model the Smlt4673 protein from residue A28 onward. AlphaFold was obtained from https://github.com/deepmind/alphafold. The script provided by the developers was used to download all required databases (software and databases were downloaded on October 18th, 2022). The AlphaFold script was run with default parameters using the “monomer_ptm” model, full databases and multiple sequence alignments constructed using all templates available at the download date. 3D structure supersposition was carried out using DaliLite.v5.1 (Holm, 2019) and cavities were analyzed with the web server CASTp (Tian et al., 2018). PyMOL v2.4.2 was used for protein structure visual analysis and figure preparation (The PyMOL Molecular Graphics System, Version 2.4.2, 2022).

## 3 Results

### 3.1 The *mla* operon in *S. maltophilia*

We first examined the genomic organization of the *mla* genes (Smlt4670-Smlt4675) in the *S. maltophilia* K279a reference genome and compared it to that previously found in other Pseudomonadota (**Supplementary Figure 1A**). BioCyc predicted that the *mla* operon is divided into two distinct transcription units: *smlt4670* (*mlaF*) and *smlt4671* (*mlaE*), and *smlt4672* (*mlaD*) to *smlt4675* (*mlaA*). However, several lines of evidence suggest that all six genes belong to the same transcription unit: (i) the *mla* genes are oriented in the same direction on the chromosome including a maximum intergenic region of 59 bp between them (*mlaE* to *mlaD*), (ii) the lack of canonical promoters between *mlaF* and *mlaA* as predicted by the PRODORIC database, (iii) the existence of a complete *mla* operon with similar organization in other species, such as *Neisseria gonorrhoeae* (Baarda et al., 2019), (iv) transcriptomic studies for *S. maltophilia* K279a in different growth phases revealed a common deregulation of the expression of these six genes (Coves et al., 2023), and (v) mapping of reads indicated the existence of a single transcription unit, although there appear to be internal promoters upstream to some CDS such as *smlt4674* (*mlaB*) and *smlt4675* (*mlaA*) (**Supplementary Figure 1B**). Taken together, these observations strongly suggest the organization of the genes of the Mla system in a single polycistronic operon in *S. maltophilia*.

### 3.2 Sequence and structural analysis of *S. maltophilia* MlaC

**Figure 1:**
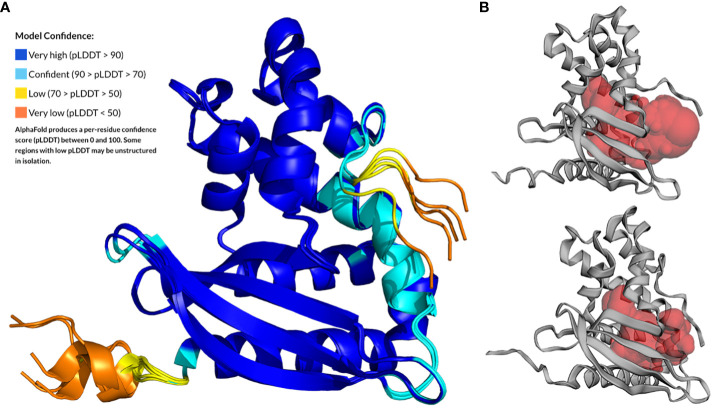
AlphaFold prediction for the 3D structure of MlaC (Smlt4673) of *S. maltophilia* from residue A28 to the last protein residue. **(A)** The five models generated by AlphaFold superimposed to the top-ranked model and colored according to the per-residue pLDDT score (Model Confidence). **(B)** CASTp-predicted ligand-binding cavity for the Smlt4673 AlphaFold models for which the cavity volume is highest (upper figure) and lowest (lower figure).

The MlaC (Smlt4673) protein of *S. maltophilia* is the soluble periplasmic PL-binding component of the Mla system. First, the primary amino acid sequence of Smlt4673 was compared with that of orthologous proteins from *P. aeruginosa*, *E. coli*, and *R. solanacearum*, whose 3D structures have already been solved (**Supplementary Figure 2**). Despite low sequence identity between the MlaC protein of *S. maltophilia* and other organisms (**Supplementary Figures 1A**, **2**), the protein structure is expected to be highly conserved as shown for other species (Yero et al., 2021). The AlphaFold prediction for the 3D structure of Smlt4673 (UniProt accession B2FP85) yielded five models with pLDDT scores between 91.9 and 89.52 (**Figure 1A**), showing great confidence in the central zone of the sequence, which aligns perfectly with the Hidden Markov Model profile that defines the Pfam family MlaC (PF05494), leaving the areas with less confidence at the extremes. A superposition of all models over the best-ranking one resulted in root-mean-square deviation (RMSD) values between 0.9Å and 1.3Å, indicating a high similarity between them (**Supplementary Table 1**). The C-terminal region was predicted as unstructured (Tunyasuvunakool et al., 2021), likely due to the presence of glycine and proline residues (Hutchinson and Thornton, 1994).

Superposition of the best-scoring model for Smlt4673 with the 3D structures of orthologous proteins whose amino acid sequences are aligned in **Supplementary Figure 2** confirmed that structure is conserved among members of this protein family (**Supplementary Figure 3**). As shown in **Supplementary Table 1**, the most similar structure is that of *P. aeruginosa*, with an RMSD of 2.3Å, 176 out of 190 superposed amino acids, and a sequence identity of 25%. Note that all structures used for this comparison were solved in complex with a lipid ligand in its hydrophobic pocket. Finally, an analysis of the ligand-binding cavity in the five AlphaFold models revealed volumes between 1777.2 and 2751.6 Å^3^ (**Figure 1B**), which were compared to the values for the already known ortholog structures in **Supplementary Table 1** (see **Supplementary Figure 4** for a surface representation of the cavity in orthologous structures). According to the models, the Smlt473 cavity would be smaller than that of the MlaC ortholog of *P. aeruginosa*, but larger than those in the other MlaC proteins used for comparison (*E. coli* and *R. solanacearum*). Amino acids and secondary structures that could contribute to the size and flexibility of the cavity and ligand binding are shown in **Supplementary Figures 3**, **5**.

The original description of the first structure solved for a MlaC protein, from *E. coli* (PDB 5UWA) (Ekiert et al., 2017), identified its fold with a transport protein domain known as NTF2. In a later study, the presence of a second domain, similar to the small helical domain of AAA+ ATPases, was identified and investigated (Yero et al., 2021). It was suggested that this domain may be responsible for the increase in cavity volume of MlaC proteins relative to other proteins with NTF2-type domains. In particular, the capacity of different MlaC orthologs to simultaneously transport two PLs was evaluated. This capacity was attributed to a bend caused by a glycine residue in the C-terminal helix and an adjacent tryptophan (G195 and W196 in 6HSY). The sequence of MlaC from *S. maltophilia* does not have either of these amino acids at these positions (E205 and L206) and the generated model displays a straight helix with no bend at this position. In fact, MlaC from *S. maltophilia* has a sequence very similar to that of *E. coli* (Q196 and L197), whose structure also shows a straight helix. However, the high presence of glycine residues down the sequence (G209, G214, P215, G217, G219) is likely to provide high flexibility to the region (Hutchinson and Thornton, 1994), as predicted by AlphaFold. This could translate in a capacity to accommodate one or two substrates, as shown for the *P. aeruginosa* protein (**Supplementary Figures 2**, **3**).

Analyzing the superposition of the known structures of the MlaC family, one recognizes the persistent presence of bulky residues inside the small helical AAA-like domain. In all but the 6HSY structure, the aromatic side chain of the residue superposed to F190 in Smlt4673 AlphaFold model (i.e., F181 in 2QGU and W181 in 5UWA) is occupying the same space. In *P. aeruginosa*, thanks to the bend produced by G195, this space is occupied by W196, which displaces F178 (the residue superposed to Smlt4673 F190) towards the interior of the domain, increasing the number of atoms housed there. Most likely, this causes the separation between the helices that form this domain in 6HSY, increasing the volume of the cavity in the NTF2 domain and enabling the binding of a second PL. Another position occupied in all cases by an aromatic residue except in 2QGU is Smlt4673 F104, which most likely would be interacting with the previously mentioned aromatic residue (**Supplementary Figures 2**, **4A, B**). On the other hand, all the structures studied show the presence of two bulky residues facing each other, marking the beginning of this domain and interacting with the PL (**Supplementary Figure 5C**).

### 3.3 Phospholipid cargo in protein MlaC of *S. maltophilia*

**Figure 2:**
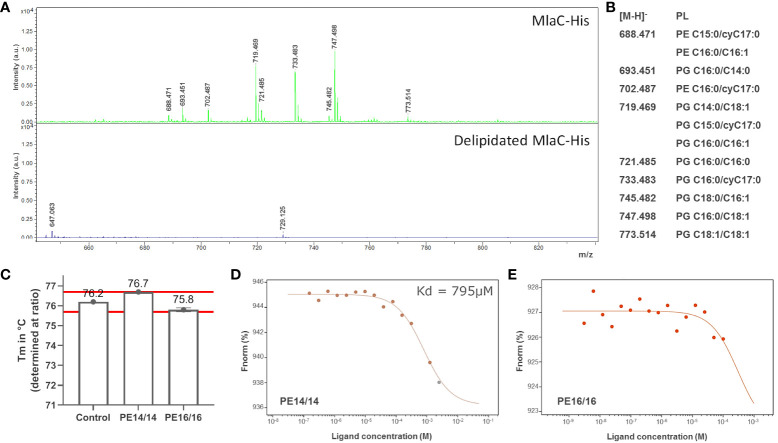
MlaC of *S. maltophilia* is a promiscuous PL-binding protein. **(A)** Determination of PL species co-purified with MlaC expressed in *E. coli* before (upper panel) and after (lower panel) delipidation of the protein by HPLC. MALDI-TOF/TOF mass spectra under denaturing conditions in negative ion mode. **(B)** Relationship of m/z values to the most probable PL species in *E. coli*. Abundant [M-H]− ions were observed for two glycerophospholipid classes: phosphatidylglycerols (PGs) and phosphatidylethanolamines (PEs). **(C)** T_m_ shift of MlaC protein with PLs, compared with protein control in 1% DMSO, as determined by nanoDSF. PE14/14 (PE C14:0/C14:0) shows a shift in the edge of significance (red lines at +/- 0.5°C from control). **(D, E)** K_d_ determination with PE14/14 and PE16/16 (PE C16:0/C16:0) by MST confirmed potential binding of PE14/14, with no evidence of binding for PE16/16, as also observed with nanoDSF. The normalized fluorescence (Fnorm%) is indicated in the y-axis.

Denaturing mass spectrometry was used to determine the lipids that were specifically bound to recombinant MlaC from *S. maltophilia* produced in the cytoplasm of *E. coli* (**Figure 2A**). Although this is clearly not the natural environment of the protein (i.e., the periplasmic space of *S. maltophilia*), several types of PLs were identified by the assay. A delipidated variant of the protein was used as a control. PLs bound by *S. maltophilia* MlaC belonged primarily to the phosphatidylglycerol (PG) and phosphatidylethanolamine (PE) types (**Figure 2B**), as previously described for other MlaC proteins. Lipid species with *m/z* of 719.5, 733.5 and 747.5 were found to be the most abundant under the assayed conditions (MALDI-TOF MS in the negative mode).

*In vitro* evaluation of the binding of delipidated MlaC to free PLs using microscale thermophoresis (MST) and nanoDSF provided the protein’s binding affinity for the specific PE variants C14:0/C14:0 and C16:0/C16:0 (**Figures 2C–E**). For C16:0/C16:0, no binding to MlaC was observed in either assay. For C14:0/C14:0, both assays showed potential binding to MlaC, albeit with a low-affinity K_d_ of 795 µM (MST).

### 3.4 The Mla system of *S. maltophilia* contributes to the maintenance of the OM barrier

**Figure 3:**
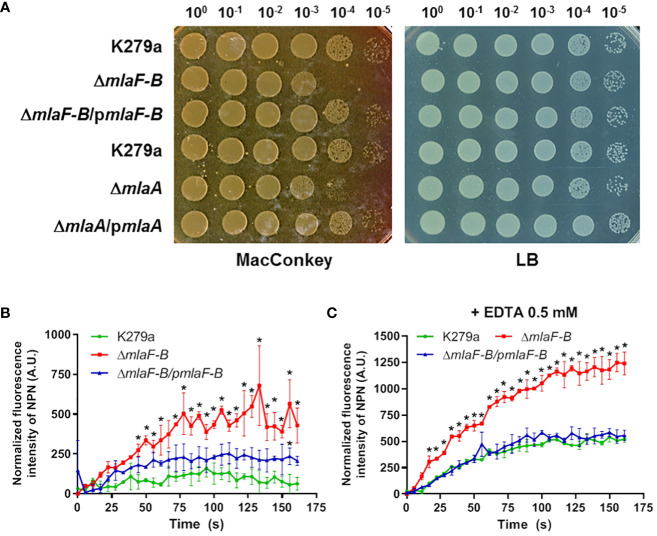
The Mla system is required to maintain the homeostasis of the OM barrier. **(A)** Plating efficiency assay on MacConkey and LB agar plates. The ten-fold dilutions are indicated above each plate. K279a Δ*mlaF-B* and Δ*mlaA* mutants were more sensitive to bile salts. Complementation of each mutant restored the wild-type phenotype. Complementation plasmids pBBR1MCS1-*mlaF-B* and pBBR1-BAD-Cm-*mlaA* are indicated as p*mlaF-B* and p*mlaA* respectively. **(B)** NPN uptake, represented by the increase in fluorescence compared to cells not treated with NPN, without any supplementation. Δ*mlaF-B* showed increased cell permeability, which was partially restored by complementation. **(C)** NPN uptake assay in the presence of 0.5 mM EDTA. Asterisks represent 2-way ANOVA with *post hoc* Bonferroni test results for each time point versus the wild-type strain (*P*<0.05).

We investigated the effects of the Mla system on OM permeability by analyzing two K279a mutant strains, one lacking the components of the *mla* operon except for *mlaA* (Δ*mlaF-B*) and the other lacking the *mlaA* gene (Δ*mlaA*). First, we showed that none of the mutants exhibited a growth defect under different standard laboratory conditions (**Supplementary Figure 6**). In order to confirm an OM-barrier defect due to an incomplete Mla system, the sensitivities of the wild-type, mutants and their complemented strains to OM stressors, such as the chelating agent EDTA, SDS, and bile salts, were measured. The mutants were fourfold more susceptible to EDTA (MIC = 0.78 mM) than the parental wild-type strain (MIC = 3.125 mM). However, no difference was observed between the mutant and wild-type cells in their susceptibility to SDS, with an MIC value of 0.04% for all strains (**Table 3**). On the other hand, plating efficiency assays in the presence of bile salts (MacConkey) showed that growth of cells lacking the Mla system was partially impaired in comparison with wild-type and complemented strains (**Figure 3A**).

**Table 3:** Antimicrobial susceptibility profile of *S. maltophilia* mutants with defects in the Mla system and complemented strains.

Antimicrobial agent	Minimum inhibitory concentration (MIC)^a^					
	K279a/pBBR1MCS1	Δ*mlaF-B*/pBBR1MCS1	Δ*mlaF-B*/p*mlaF-B*	K279a/pBBR1-BAD	Δ*mlaA*/pBBR1-BAD	Δ*mlaA*/p*mlaA*
Sulfonamides						
Trimethoprim-sulphamethoxazole	0.25	0.25	0.25	0.25	0.25	0.25
Polypeptides						
Colistin	4	**1**	4	4	**1**	2
Carbapenems (beta-lactam)						
Imipenem	128	128	128	128	128	128
Meropenem	8	8	8	8	8	8
Cephalosporins (beta-lactam)						
Ceftazidime	2	2	2	2	2	2
Penicillins (beta-lactam)						
Piperacillin-tazobactam	16	16	16	16	16	16
Ticarcillin-clavulanic	1	1	1	1	1	1
Aminoglycosides						
Tobramycin	32	32	32	32	32	32
Amikacin	8	8	8	8	8	8
Streptomycin	32	32	32	32	32	32
Chloramphenicol						
Chloramphenicol	128	128	128	128	128	128
Tetracyclines						
Tetracycline	16	16	16	16	16	16
Minocycline	1	**0.25**	1	1	**0.25**	1
Tigecycline	4	4	4	4	4	4
Fluoroquinolones						
Ciprofloxacin	8	8	8	8	8	8
Levofloxacin	4	4	4	4	4	4
Ofloxacin	4	4	4	4	4	4
Norfloxacin	32	32	32	32	32	32
Other membrane-damaging agents						
EDTA (in mM)	3.125	**0.78**	3.125	3.125	**0.78**	3.125
SDS (in %)	0.04	0.04	0.04	0.04	0.04	0.04

a Minimum inhibitory concentration (MIC) determined by the broth microdilution method. The MIC is expressed in μg/mL, except for the EDTA and SDS agents. MICs were confirmed by two or three independent replicates and MIC differences greater than 2-fold with respect to the wild type strain were considered significant (indicated in bold). Complementation plasmids pBBR1MCS1-mlaF-B and pBBR1-BAD-Cm-mlaA are indicated as p*mlaF-B* and p*mlaA* respectively. For the MIC of EDTA and SDS, the wild type and mutant strains were not transformed with the empty vectors.

To investigate whether the deletion of components of the Mla system affects antibiotic resistance, the susceptibility of all strains to a broad spectrum of antibiotics was analyzed. As shown in **Table 3**, both the *mlaF-B* and *mlaA* mutants showed increased susceptibility to colistin and minocycline among all antibiotics tested. The complemented strains showed restored MIC values. However, the sensitivity to the rest of the antibiotics remained unchanged. After these results and the evidence that both mutants showed the same susceptibility phenotypes, we decided to proceed with the mutant strain Δ*mlaF-B*.

The uptake rate of 1-*N*-phenylnapthylamine (NPN) was also measured. The NPN probe fluoresces strongly in PL environments but only weakly in aqueous environments, and a damaged OM should be more permeable to hydrophobic substances such as NPN. The increase in fluorescence levels was significantly higher in the Δ*mlaF-B* strain (*P*<0.05) compared to the parental wild-type strain in the presence of 10 µM NPN (**Figure 3B**), indicating a compromised integrity of the OM, partially restored by *in trans* complementation of Δ*mlaF-B* with pBBR1MCS1-*mlaF-B*. To demonstrate that the *mla*-defective strain was more sensitive to external stressors, EDTA was added at a subinhibitory concentration of 0.5 mM, which resulted in more pronounced differences when comparing the wild-type strain and the *mla* mutant (**Figure 3C**). EDTA at this concentration did not affect the growth of the strains used in the assay (**Supplementary Figure 6**).

### 3.5 The absence of the Mla system affects the cell morphology of *S. maltophilia* in the presence of EDTA

**Figure 4:**
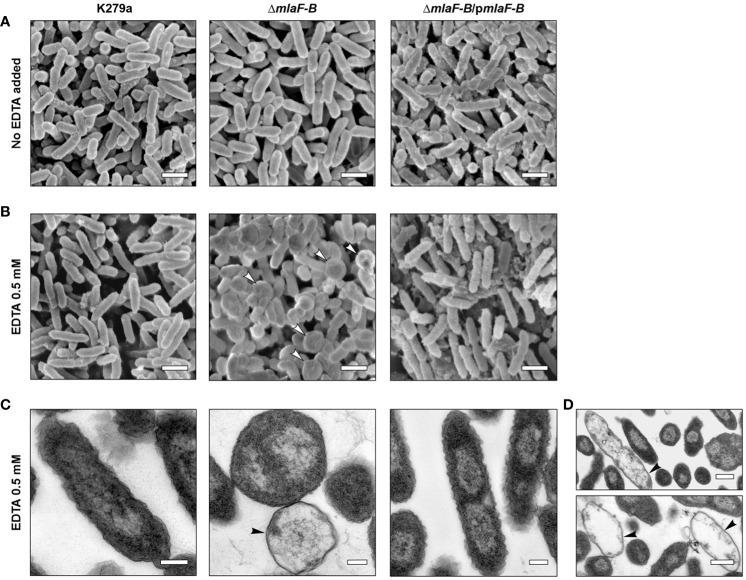
Cells with deleted Mla components showed an altered cell morphology in the presence of EDTA. SEM analysis of bacterial cells without EDTA treatment **(A)** or treated with 0.5 mM EDTA **(B)**. Putative bacterial ghost cells are indicated by white arrowheads. Empty cell envelopes without cytoplasmic and nuclear contents as shown by TEM and marked with black arrowheads **(C, D)**. The scale bars in panels **(A, B)** represent 1 µm, and the scale bars in panels **(C, D)** correspond to 0.2 µm and 0.5 µm, respectively. Complementation plasmid pBBR1MCS1-*mlaF-B* is indicated as p*mlaF-B*.

To further investigate the effects that the mutation in the Mla system could have on *S. maltophilia* cells, we examined morphological changes in cell structure using electron microscopy. In a first step, samples from cell cultures of the wild-type strain, the mutant Δ*mlaF-B* and the complemented strain were examined by scanning electron microscopy (SEM). Under normal culture conditions, i.e., without addition of the chelating agent EDTA, the cells exhibited normal morphology with the characteristic rod-like shape of an *S. maltophilia* bacterium (**Figure 4A**). Despite a defective Mla system, no obvious morphological or structural differences were observed in Δ*mlaF-B* cells in the absence of EDTA (**Figure 4A**), suggesting that either the loss of components of the Mla system is not critical for maintaining the structural integrity of the membrane or that other systems are involved in compensating for this deficiency. Previous studies had also shown that cell structure was not affected in MlaA mutants of *N. gonorrhoeae* (Baarda et al., 2019) and *Haemophilus influenzae* (Fernández-Calvet et al., 2018).

In contrast, after treatment with EDTA at the subinhibitory concentration of 0.5 mM, mutant cells showed important morphological disorders. The Δ*mlaF-B* cells adopted a more spherical shape with smaller size in the presence of EDTA, indicating perturbations in the structural integrity of the cell envelope (**Figure 4B**). The detrimental effects on the integrity of the bacterial cell envelope were further confirmed by the detection of bacterial ghost cells with irregular shapes in SEM and TEM (**Figures 4B–D**), with the TEM images showing in particular empty cell envelopes devoid of cytoplasmic and nuclear contents (**Figures 4C, D**). Although the observed characteristics of the mutant strain upon contact with 0.5 mM EDTA indicated a decrease in cell viability, no effect of this concentration on the growth rate of the mutant strain could be detected (**Supplementary Figure 6**).

### 3.6 Impaired biofilm formation by the Mla system mutant both in mono- and co-culture with *P. aeruginosa*

**Figure 5:**
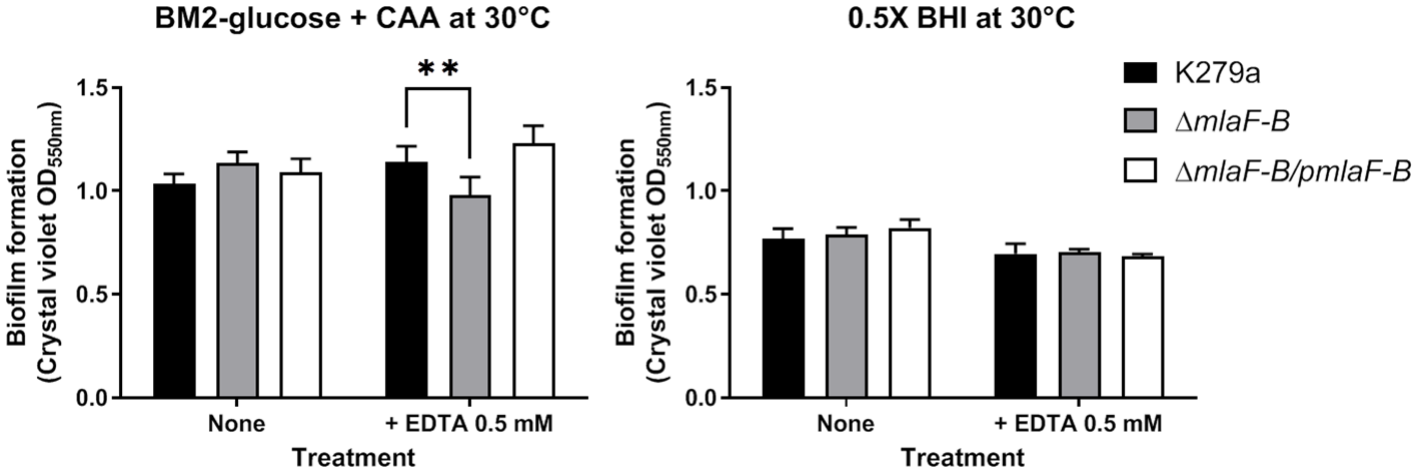
Biofilm formation is reduced in the K279a Δ*mlaF-B* mutant when grown in modified BM2 minimal media. Cells were grown in a polystyrene microtiter plate in BM2-glucose minimal medium supplemented with casamino acids (CAA) or 0.5 × BHI at 30°C and without and with 0.5 mM EDTA. The amount of each biofilm was quantified by crystal violet staining (OD_550_ value) after 24 hours of incubation under static conditions. Complementation plasmid pBBR1MCS1-*mlaF-B* is indicated as p*mlaF-B*. Data are means ± SD (n = 6). Two-way ANOVA with *post hoc* Bonferroni’s multiple comparison test was used to determine the significance of the data between groups (**P < 0.01).

We studied biofilm formation in microtiter plate under static conditions using K279a wild-type, mutant, and complemented strains grown in 0.5 × BHI or a modified BM2-glucose minimal medium (**Figure 5**). The amount of biofilm formed by the Δ*mlaF-B* mutant was lower than that of the wild-type strain in the presence of EDTA (0.5 mM), but only when the cells were grown in minimal medium (**Figure 5**). EDTA at this concentration did not affect the planktonic growth of the strains under the same conditions used for biofilm formation (**Supplementary Figure 6**). To confirm the unique role of EDTA in biofilm reduction in the mutant strain, we also showed that production of both exopolysaccharide and the quorum sensing signal DSF (diffusible signal factor) were not affected in this strain (**Supplementary Figure 7**). In *S. maltophilia*, exopolysaccharide biosynthetic genes are necessary for biofilm production (Huang et al., 2006) and DSF positively regulates biofilm formation (Alcaraz et al., 2019).

**Figure 6:**
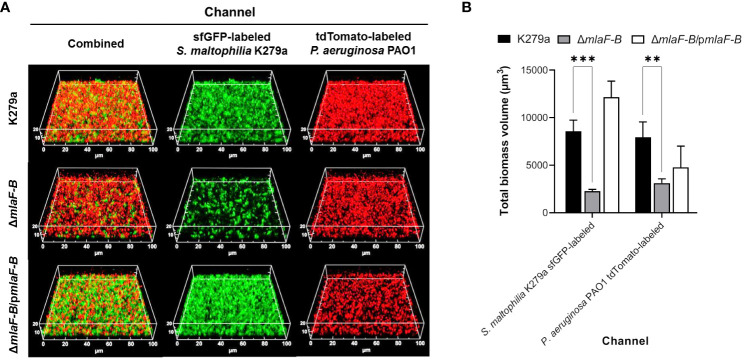
Mutation in the Mla system of *S. maltophilia* reduces the biomass of mixed biofilm with *P. aeruginosa*. **(A)** CLSM images of dual species biofilms of sfGFP-labelled *S. maltophilia* K279a strains (green) and *P. aeruginosa* PAO1::tdTomato (red) formed after incubation for 72 hours in 0.5 × BHI at 30°C. Complementation plasmid pBBR1MCS1-*mlaF-B* is indicated as p*mlaF-B*. **(B)** Quantification of fluorescence signals derived from CLSM 3D images. Data are means ± SD (n = 3). Two-way ANOVA with *post hoc* Bonferroni’s multiple comparison test was used to determine the significance of the data between groups (**P < 0.01; ***P < 0.001).

In addition, for formation of mixed biofilms, sfGFP-labelled strains of *S. maltophilia* were incubated in 8-well µ-Slide chambers with wild-type, tdTomato-labelled *P. aeruginosa* strain PAO1 (**Figure 6**). We chose the BHI medium because it has proven to be the most robust medium for studying *in vitro* biofilm formation of a wide range of bacteria. We had previously shown that *S. maltophilia* and *P. aeruginosa* do not compete with each other under standard laboratory culture conditions and that *S. maltophilia* strains are not sensitive to compounds exogenously produced by *P. aeruginosa* (**Supplementary Figure 8**). As observed in the *S. maltophilia* monospecies biofilms with EDTA, the mutant Δ*mlaF-B* also formed less biofilm in the presence of *P. aeruginosa*, although in this case biofilm formation was even more impaired (**Figure 6**). In the competition assays, we observed that when the mixed culture conditions were static (without shaking), the planktonic population of *S. maltophilia* was reduced by one log order, but this was equally the case for all strains examined (**Supplementary Figure 8D**). Interestingly, biomass quantification of the 72-hours biofilms showed that *P. aeruginosa* cells were also affected in the mixed biofilm in the presence of the mutant Δ*mlaF-B* (**Figure 6B**), which seems to indicate a bidirectional effect.

## 4 Discussion

The Mla system in Gram-negative bacteria is involved in the transport of PLs between the outer and inner membranes, maintaining OM asymmetry, where PLs are primarily found in the inner leaflet, thus avoiding the formation of misplaced PL patches in the OM outer leaflet (Malinverni and Silhavy, 2009; Henderson et al., 2016). However, this is not the only mechanism involved in OM lipid homeostasis, and this is likely the reason of the weak phenotypes observed in mutants with defects in the Mla system, including those shown in this work. For example, the phospholipase PldA of *E. coli* degrades mislocalized PLs for transport of the fatty acids to the cytoplasm for their recycling (Snijder and Dijkstra, 2000; Malinverni and Silhavy, 2009). In *S. maltophilia* K279a, the phospholipase A Smlt3218 is an ortholog of *E. coli* PldA (53% similarity, 89% coverage), and may partly compensate for the lack of the Mla system in the mutant. In species that lack PldA, such as *P. aeruginosa*, a new compensatory system named MlaZ/MlaY has recently been discovered (Guest et al., 2023). This system consists of a lipase and a MlaA-like protein and coordinates the removal of misplaced PL in the OM. A similar system has not been detected by sequence homology in *S. maltophilia*. Although several systems exist to control the lipid composition of the OM, we provide evidence of some physiological consequences of the deletion of the Mla system in *S. maltophilia*.

We have shown that the Mla system helps in making the membrane less permeable to harmful substances, as has been observed for other Gram-negative bacteria (Malinverni and Silhavy, 2009; Bernier et al., 2018; Fernández-Calvet et al., 2018; Baarda et al., 2019; Kamischke et al., 2019; Yero et al., 2021; de Jonge et al., 2022), and that this role affects physiological aspects of these bacteria and their interaction with other microorganisms. Among the interfering agents evaluated, the action of divalent-ion chelators stood out as the most sensitive to the Mla function. Antibacterial agents such as bile salts, for example, are known to disrupt bacterial membranes and chelate iron and calcium (Urdaneta and Casadesús, 2017). Regarding antibiotic resistance phenotypes, the lack of the Mla system in *S. maltophilia* increased only the susceptibility to colistin and minocycline. Previous studies in *P. aeruginosa* have shown that mutations in the MlaC system have minimal or no impact on those antibiotics to which bacterial strains are equipped with specific mechanisms that confer high-level resistance (Yero et al., 2021). *S. maltophilia* are intrinsically resistant to multiple antibiotics, including the K279a strain used in this work. However, this strain can be considered susceptible to minocycline and it displays a low resistance to colistin compared to other strains (Yero et al., 2020). Furthermore, the role of the MlaC system in antibiotic resistance is clearly associated with the PL composition of the OM. It is known that the proportion of the different PL classes in the OM can alter the susceptibility of bacteria to certain antibiotics, e.g. by forming PL patches or changing the structure of integral membrane proteins such as efflux pumps (Bernal et al., 2007; Epand et al., 2008).

The soluble periplasmic PL-binding component of the Mla system is the MlaC protein. The role of this protein in PL transport has been extensively discussed in the literature (Ercan et al., 2019; Hughes et al., 2019; Yeow et al., 2023), and it has already been shown to have affinity for several PL classes (Yero et al., 2021). The low affinity for binding free PL shown in this work is consistent with the results obtained by Hughes et al. (2019) and may be related the fact that MlaC requires helper proteins to some extend for PL uptake and release. MlaC passes PLs between MlaA and MlaD, acting as a transporter in the periplasm (Tang et al., 2021), and both proteins appear to be separately required to pass PLs to MlaC (Thong et al., 2016). On the other hand, structural studies and molecular dynamics simulations on apo and PL‐bound MlaC show that the binding pocket can be in an open or closed state and suggest that the apo conformation does not easily shift to the open conformation (Huang et al., 2016; Hughes et al., 2019). In addition, our work and common sense suggest that the affinity for different PLs is tailored to the PL composition of each species. This affinity is governed by the hydrophobic chains of the fatty acids and not by the nature of the polar head of the PLs, since this is exposed to the medium (Huang et al., 2016; Yero et al., 2021). Although the MST and nanoDSF experiments only indicate a potential binding with PE C14:0/C14:0, the preference for PLs with 14-carbon-atom fatty acids deserves to be discussed. Unlike other bacterial families, the most abundant fatty acid in *S. maltophilia* is 13-methyl-tetradecanoic acid (iso-15:0) (Huedo et al., 2015; Coves et al., 2023), a branched-chain saturated fatty acid. Our MlaC 3D structure predictions indicate a potential evolution of this system to accommodate branched PLs fatty acids. The co-evolution of the Mla system in Gram-negative bacteria with the PL composition of the membrane indeed deserves further investigation.

In this work, we have also shown that the cooperation between *S. maltophilia* and *P. aeruginosa* in the formation of mixed biofilms depends, in part, on *S. maltophilia* membrane permeability, mediated by the Mla system. Previous *in vitro* studies have shown that both microorganisms are able to live in integrated communities forming polymicrobial biofilms (Ryan et al., 2008; McDaniel et al., 2020; Alio et al., 2023), although this cooperation may be strain specific (Pompilio et al., 2015). The cooperation seen between *P. aeruginosa* and *S. maltophilia* is primarily attributed to the QS systems of both species. The synthesis and secretion of DSF by *S. maltophilia* affects the expression of stress-response factors in *P. aeruginosa*, such as regulatory systems controlling resistance to polymyxin and antimicrobial peptides (Ryan et al., 2008). On the other hand, *S. maltophilia* cells can respond to acyl-homoserine lactone (AHL) signals secreted by *P. aeruginosa* that affect their motility (Martínez et al., 2015). Furthermore, as for other microorganisms, alginate produced by *P. aeruginosa* could provide mechanical or chemical protection for *S. maltophilia* in mixed biofilms (Pompilio et al., 2015; McDaniel et al., 2020). Surprisingly, expression of genes of the *mla* operon in *S. maltophilia* K279a was upregulated when cells were in the exponential phase of growth compared to the stationary phase (Coves et al., 2023). This could indicate that this system is controlled by cell density-dependent mechanisms, such as quorum sensing. In *S. maltophilia* many virulence features, such as extracellular enzymes, bacterial motility and biofilm formation, are finely controlled by its DSF-dependent quorum sensing system (Huedo et al., 2018). However, DSF production in the Mla system mutant was not affected, suggesting that the regulation of the expression of these genes is under the control of other cellular mechanisms that are modulated in the stationary phase.

The role of the Mla system in protecting cells from divalent-ion chelating agents could explain the results obtained for both axenic and polymicrobial biofilms. Divalent cations stabilize the biofilm as they contribute to cross-linking the matrix through electrostatic interactions (Cavaliere et al., 2014). The observation of EDTA-induced impairment of biofilm formation in the Mla mutant strain illustrates this relation. On the other hand, extracellular DNA and exopolysaccharides, the main components of the biofilm matrix in *P. aeruginosa*, can act as cation chelators due to their highly anionic character (Horsman et al., 2012; Wilton et al., 2016), which may explain the abrupt decrease in biofilm formation by *S. maltophilia* in the presence of *P. aeruginosa*. Besides chelators, the Mla system of *S. maltophilia* may help protect this species from other known harmful effectors of *P. aeruginosa*. For example, *P. aeruginosa* secretes small molecules that can permeabilize bacterial membranes (Radlinski et al., 2017; Orazi et al., 2019). In addition, many other studies have shown that *P. aeruginosa* is able to displace other microorganisms in co-cultures *in vitro* and *in vivo* (Gomes-Fernandes et al., 2022), mainly through the action of exoproducts such as the QS signal 2-heptyl-4-hydroxyquinoline-*N*-oxide (HQNO), siderophores and other antimicrobial compounds that interfere with the growth of other species by competing for the availability of oxygen or affecting the respiratory chain (Biswas et al., 2009; Abisado et al., 2018). The Mla system in *S. maltophilia* may help to prevent the entry of these interfering molecules into cells while regulating the diffusion of hydrophobic heterologous QS autoinducers. It has been postulated that the Mla system contributes to the regulation of glycerophospholipid bilayer composition in the OM through which lipophilic molecules can pass (de Jonge et al., 2022; Guest et al., 2023). An increased diffusion of certain molecules in the *S. maltophilia* mutant could explain the reduction seen in biomass of *P. aeruginosa* in the mixed biofilm, although this requires further investigation.

The results of this work reinforce the idea that the Mla system could be an interesting target for new antimicrobial strategies. For example, a drug aimed at inhibiting this system could mimic the known synergistic effects of EDTA with antibiotics. EDTA in combination with antibiotics of various classes or disinfectants has been shown to be effective against clinical strains of *S. maltophilia* or for the decontamination of medical devices (Passerini de Rossi et al., 2012; Anari et al., 2022). An antimicrobial therapy based on the MlaC transporter is also very attractive to eradicate polymicrobial biofilms formed by Gram-negative pathogens. Although the incidence is very low, there are reports of patients simultaneously infected with *S. maltophilia* and *P. aeruginosa* (Wainwright et al., 2009; Yin et al., 2017). This co-infection appears to have a synergic effect on the mortality and clinical outcome of pneumonia patients (Yin et al., 2017), which has also been suggested in experimental respiratory infections of mice (McDaniel et al., 2020).

## Data availability statement

The original contributions presented in the study are included in the article/**Supplementary Material**. Further inquiries can be directed to the corresponding authors.

## Author contributions

XC: Investigation, Writing – original draft, Writing – review & editing, Conceptualization. UM: Investigation, Writing – original draft, Writing – review & editing. OC: Investigation, Writing – original draft, Writing – review & editing, Software. PH: Investigation, Writing – review & editing. MB: Investigation, Writing – review & editing. AG: Investigation, Writing – review & editing. IK: Investigation, Writing – review & editing. WS: Writing – review & editing, Supervision. US: Supervision, Writing – review & editing. IG: Conceptualization, Investigation, Supervision, Writing – review & editing. XD: Supervision, Writing – review & editing, Conceptualization, Investigation. DY: Writing – review & editing, Conceptualization, Investigation, Writing – original draft.
