# Supplementary material for: The Mla system and its role in maintaining outer membrane barrier function in Stenotrophomonas maltophilia
Source: Front Cell Infect Microbiol. 2024 Feb 26;14:1346565. doi: 10.3389/fcimb.2024.1346565 (PMC10925693; doi:10.3389/fcimb.2024.1346565)
Supplement: Supplementary file 1 [file DataSheet_1.pdf]

SUPPLEMENTARY INFORMATION

The Mla system and its role in maintaining outer membrane barrier function in *Stenotrophomonas maltophilia*

Xavier Coves[1,2], Uwe Mamat[3], Òscar Conchillo-Solé[1,2], Pol Huedo[1,2], Marc Bravo[1,2], Andromeda-Celeste Gómez[1,2], Ines Krohn[4], Wolfgang R. Streit[4], Ulrich E. Schaible[3], Isidre Gibert[1,2], Xavier Daura[1,5,6] and Daniel Yero[1,2]

[1]Institut de Biotecnologia i de Biomedicina (IBB), Universitat Autònoma de Barcelona (UAB), 08193 Cerdanyola del Vallès, Spain. [2]Departament de Genètica i de Microbiologia, Universitat Autònoma de Barcelona (UAB), 08193 Cerdanyola del Vallès, Spain. [3]Cellular Microbiology, Priority Research Area Infections, Research Center Borstel, Leibniz Lung Center, Leibniz Research Alliance INFECTIONS, 23845 Borstel, Germany. [4]University of Hamburg, Institute of Plant Science and Microbiology, Department of Microbiology and Biotechnology, 22609 Hamburg, Germany. [5]Catalan Institution for Research and Advanced Studies (ICREA), 08010 Barcelona, Spain. [6]Centro de Investigación Biomédica en Red de Bioingeniería, Biomateriales y Nanomedicina, Instituto de Salud Carlos III, 08193 Cerdanyola del Vallès, Spain.

*Correspondence: Xavier Daura, Xavier.Daura@uab.cat, and Daniel Yero, Daniel.Yero@uab.cat

Table S1: *S. maltophilia* MlaC structural models by AlphaFold and their comparison with other known structures.[a]

Structure	Sequence	AF pLDDT	No. resid.	DALI lali	DALI RMSD	%id	Volume	Cavity's resid.	Cavity's atoms	Farthest dist.
0_relax_4	Smlt4673-B2FP85	91.9	193	193	-	100	2751.61	49	154	30.14
1_relax_3	Smlt4673-B2FP86	91.25	193	193	0.9	100	2410.67	51	151	32.92
2_relax_2	Smlt4673-B2FP87	89.98	193	193	0.7	100	2439.44	47	154	29.66
3_relax_1	Smlt4673-B2FP88	89.81	193	193	1.2	100	2312.66	48	142	28.34
4_relax_5	Smlt4673-B2FP89	89.52	193	191	1.3	100	1777.17	47	135	27.6
AF_average (stdev)	Smlt4673-B2FP89	90.49 (1.03)	193 (0)	192.5 (1)	1.03 (0.28)	100 (0)	2338.31 (354.2)	48.4 (1.67)	147.2 (8.41)	29.73 (2.05)
6HSY_A	Q9HVW4_PSEAE	-	190	176	2.3	25	3035.24	57	191	30.41
2QGU_A	Q8XV73_RALSO	-	179	170	2.5	20	1332.26	31	101	25.99
5UWA_A	P0ADV7|MLAC_ECOLI	-	185	170	2.9	26	1444.33	43	118	28.24

[a]**Notes:** For fair comparison, 6HSY is presented without hydrogens (like the other structures available). Table columns description: Structure ID and sequence source (containing Uniprot code). AlphaFold (AF) pLDDT score. Number of residues in the structure. Number of superposed residues and RMSD as reported by DALI lite. Sequence identity with respect to *S. maltophilia* sequence (%id). CASTp Connolly's volume and number of residues and atoms in the cavity. Distance between the two atoms in the cavity farthest from each other. All distances in Angstrom (Å) and Volume in $Å^3$. Mean and standard deviation (stdev) lines for AF models are underlined.

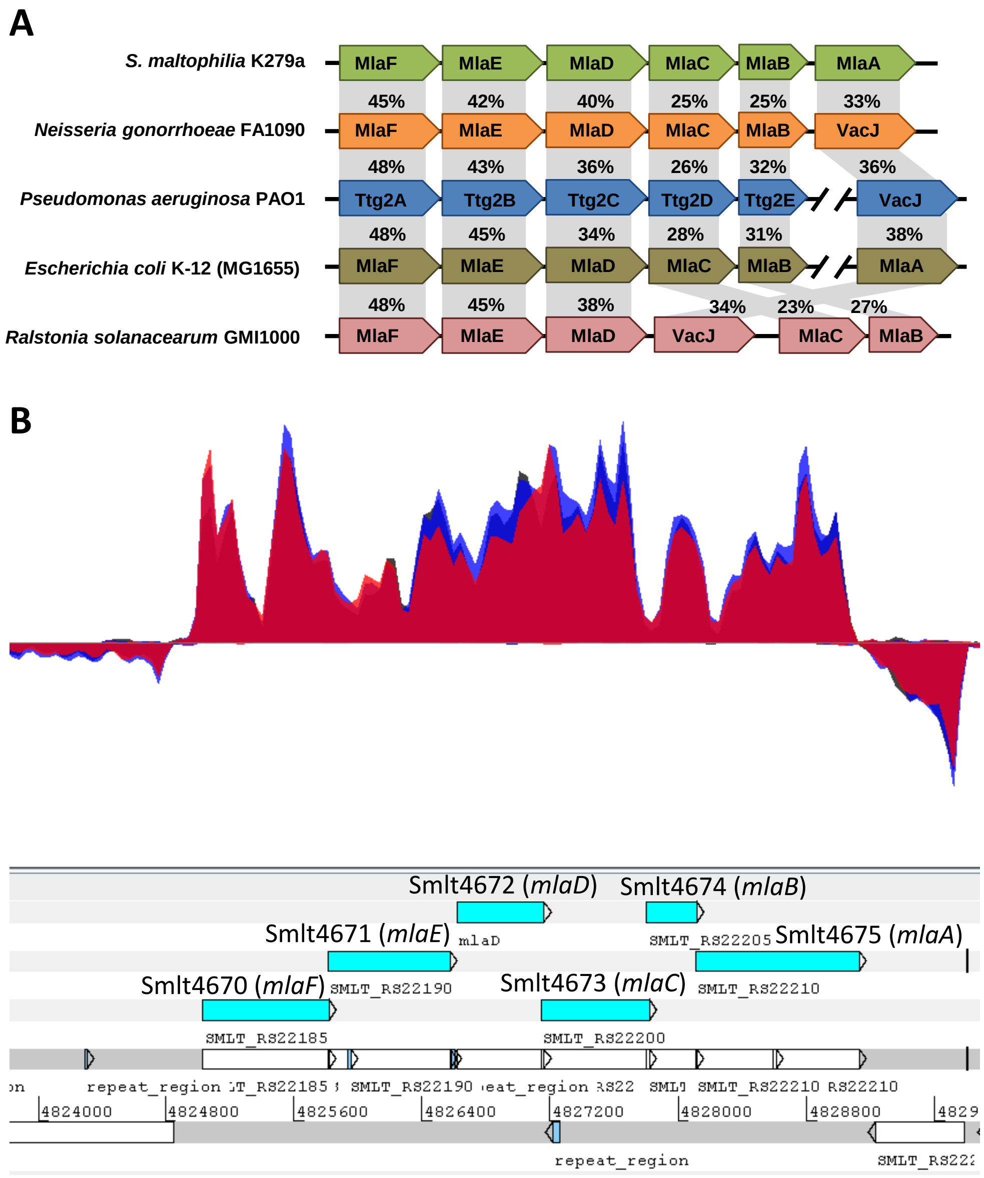

Figure S1. Structure of the *mla* operon in *S. maltophilia*. **(A)** Comparison of the genomic organization of the *mla* genes in *S. maltophilia* with those of other Gram-negative bacteria based on representative NCBI genomes. The amino acid identities (%) with respect to the orthologous sequences in *S. maltophilia*, which were determined by Clustal Omega, are shown above each gene. **(B)** Validation of the structure of the operon comprising CDS for Smlt4670 to Smlt4675 using transcriptome data. RNA-seq data (GEO GSE206442) from a previously published work (PMC10304680) was mapped to the K279a chromosome (NCBI NC_010943.1) and visualized using the Artemis genome browser. The new locus tags are indicated below each gene of this chromosomal segment, and only for those genes of the *mlaFEDCBA* operon the old locus tags are also indicated. For this analysis, RNA-seq data from RNA samples collected from cultures in exponential growth was used. Strand-specific coverage plots based on read alignments are shown in the upper panel and the genome annotation below. Each colour in the graph represents a triplicate sample.

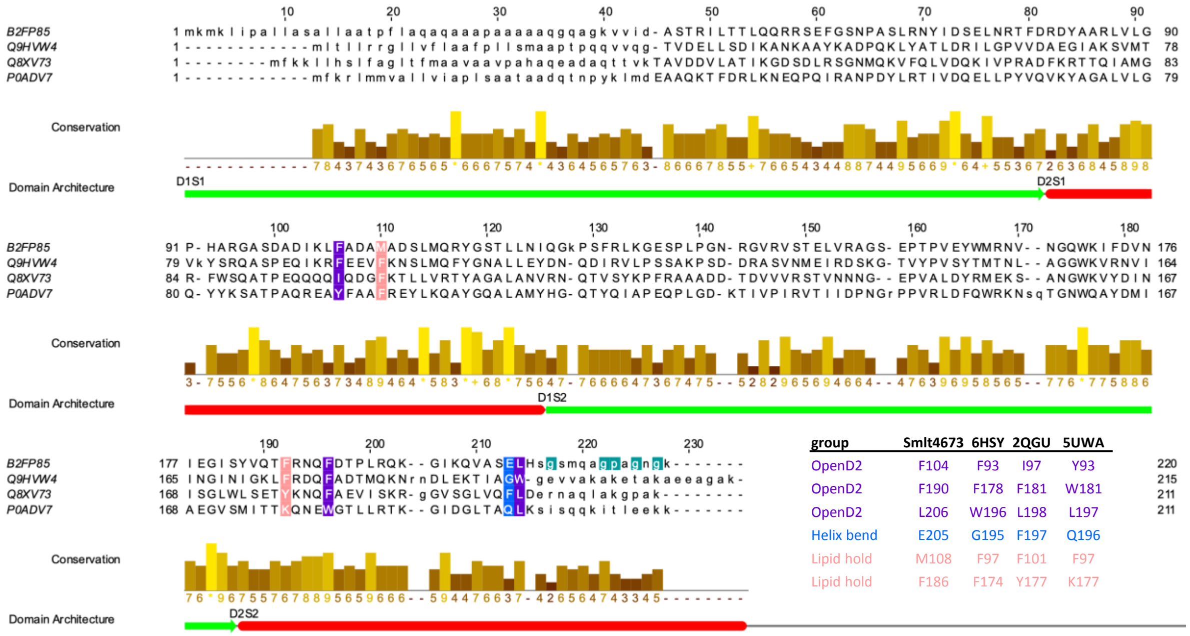

Figure S2: Multiple alignment of primary amino acid sequences of *S. maltophilia* MlaC (Smlt4673) and representative orthologous MlaC sequences from organisms with experimentally solved structures (Chain A of 6HSY, 5UWA, 2QGU). Protein sequences for *S. maltophilia* (B2FP85), *P. aeruginosa* (Q9HVW4), *E. coli* (P0ADV7) and *R. solanacearum* (Q8XV73) were obtained from the Uniprot database (https://www.uniprot.org/). Residues involved with the increased cavity volume are shown in purple (group OpenD2), residues involved in the bend/straight C-terminal helix are shown in blue (Helix bend group), residues involved with the increased cavity volume and at the same time contact with the lipid are shown in Salmon (group Lipid Hold), and residues putatively responsible for the predicted unstructured C-terminal region of *S. maltophilia* MlaC are coloured green (G209, G214, P215, G217, G219). Unaligned residues are shown in lower case letters. The degree of conservation for each position in the alignment is shown as a bar chart. The domain architecture, which consists of two segmented, non-contiguous domains, is represented by a green line for the area of alignment belonging to the segments of the first domain (NTF2 domain) and a red line for the segments of the second one (AAA+ ATPases small helical like domain).

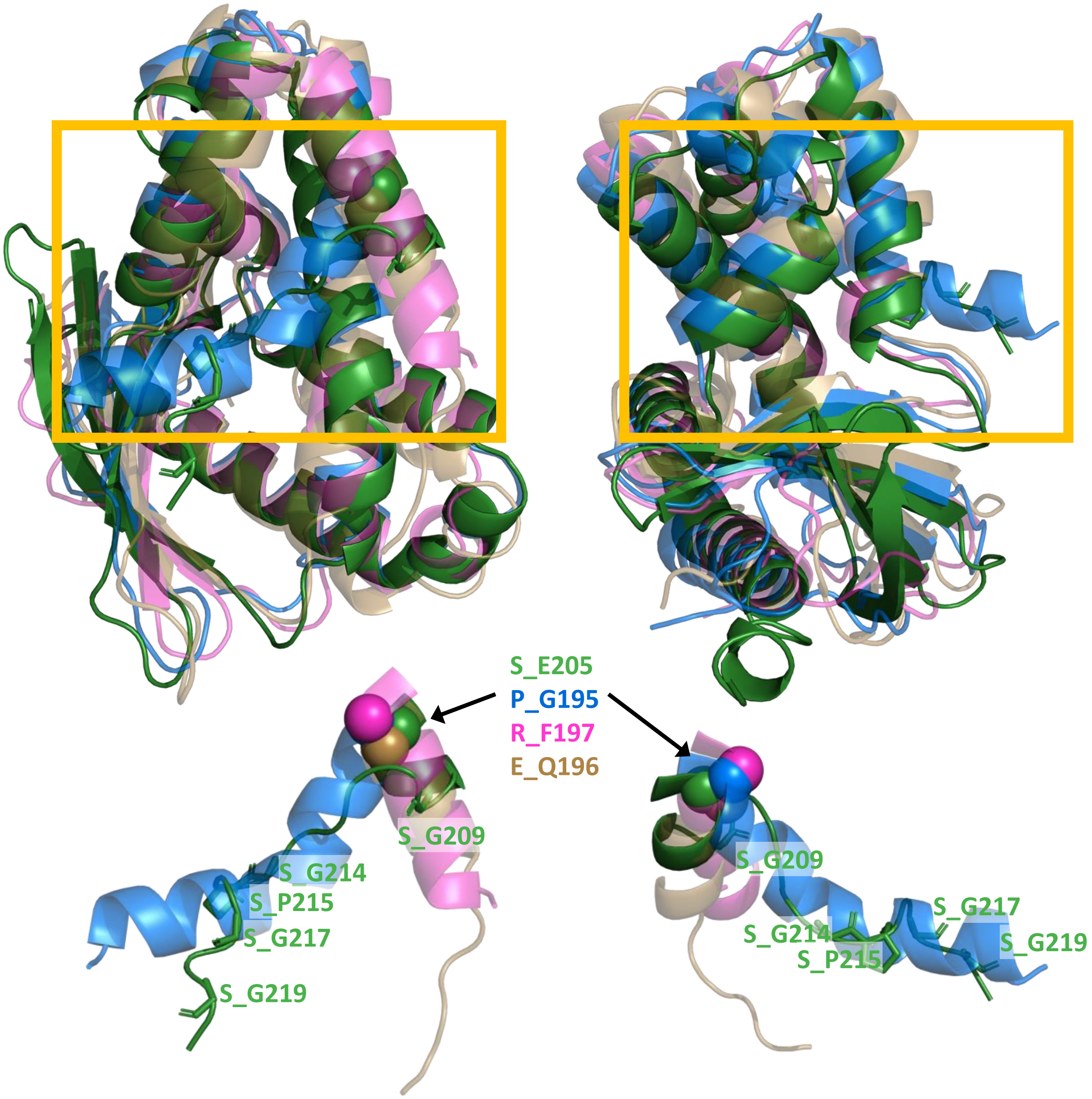

Figure S3: Two different views of the superposition of the best-scoring AlphaFold model for Smlt4673 (green forest) and the experimentally solved 3D structures for the orthologous protein of *P. aeruginosa* (PDB 6HSY, marine blue), *R. solanacearum* (2QGU, light magenta) and *E. coli* (5UWA, brown sand). The Calpha atoms of residues responsible for the C-terminal bend/straight helix are depicted as spheres (E205 in Smlt4673, G195 in 6HSY, F197 in 2QGU, Q196 in 5UWA). Figures at the bottom show a detailed view of the C-terminal residues (located in the boxed areas of the overall figures); the residues responsible of the predicted unstructured region in *S. maltophilia*'s MlaC are depicted in sticks (G209, G214, P215, G217, G219). The residues labelled in the figures indicate the first letter of the corresponding species.

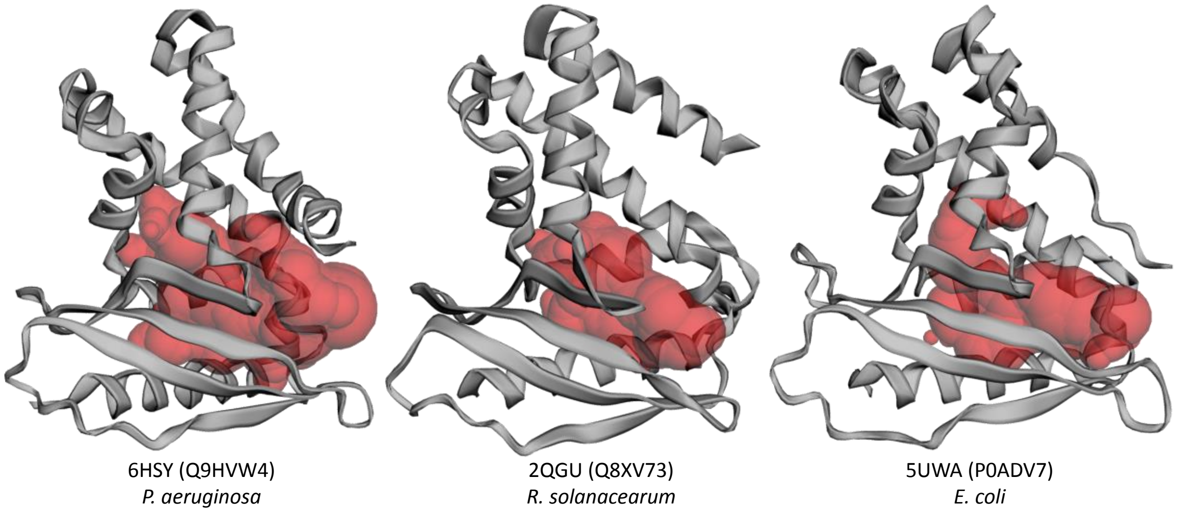

Figure S4: Surface representation of the cavity calculated in different MlaC orthologous structures (see Table S1 for volume data). The PDB code and the Uniprot identifier (in parentheses) are indicated for each species.

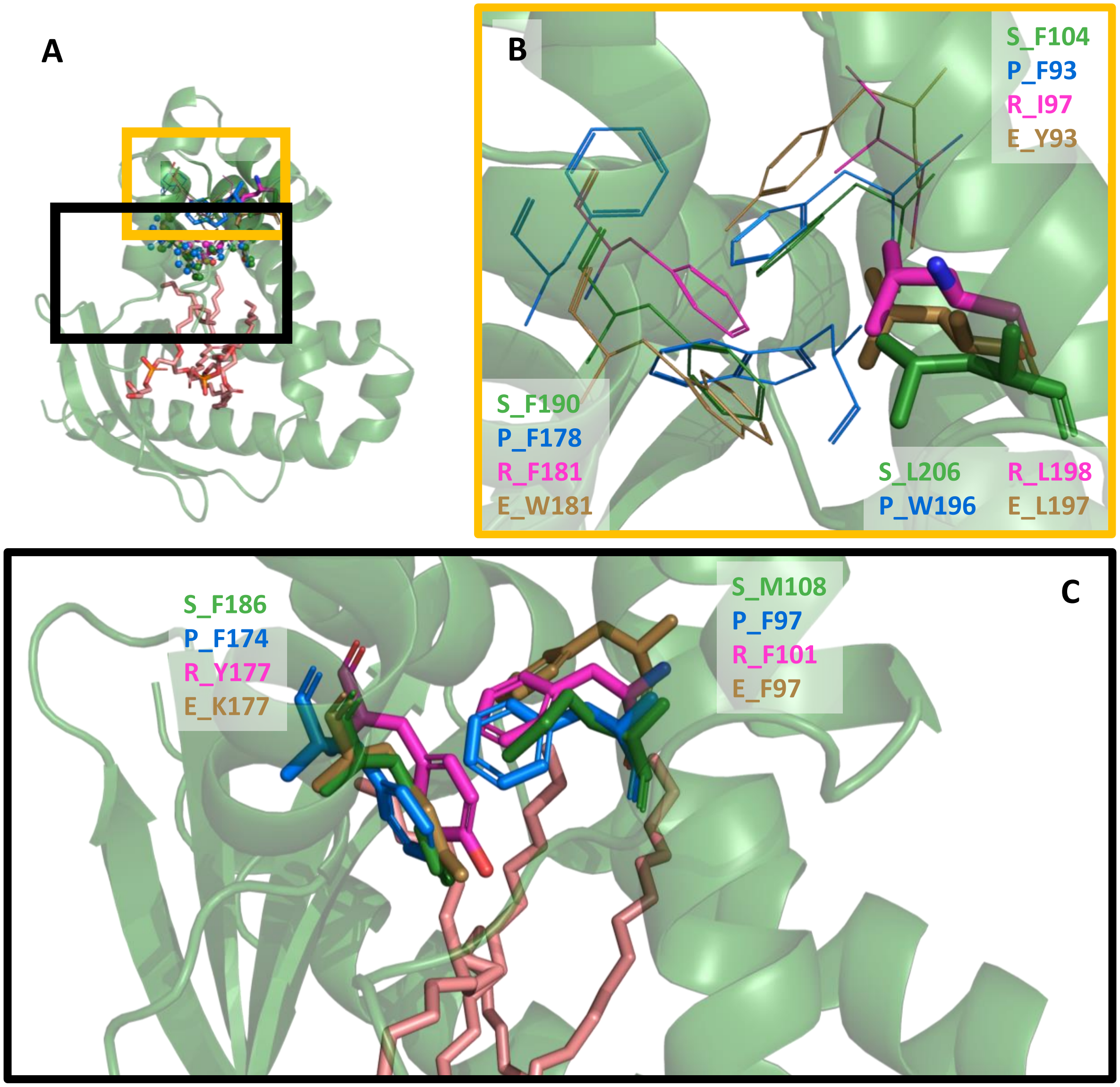

Figure S5: Residues involved in keeping the cavity volume. **(A)** Overall view of the predicted structure for Smlt4673 with the phospholipids seen inside the cavity of the ortholog structure from *P. aeruginosa* (pale pink). Critical residues from all structures used for comparison in Figures S2 and S3 are shown. **(B)** Detail of the residues involved in the increased cavity volume (group OpenD2); in sticks residues superposed to *P. aeruginosa* W196 for the other structures, all other residues in wire. **(C)** Detail of the residues that perform the same function and also contact the lipid in the cavity. In all panels, dark green residues correspond to Smlt4673, marine blue residues to the 6HSY model (*P. aeruginosa*), light magenta residues to the 2QGU model (*R. solanacearum*) and light brown residues to the 5UWA model (*E. coli*). The residues labelled in the figures indicate the first letter of the corresponding species.

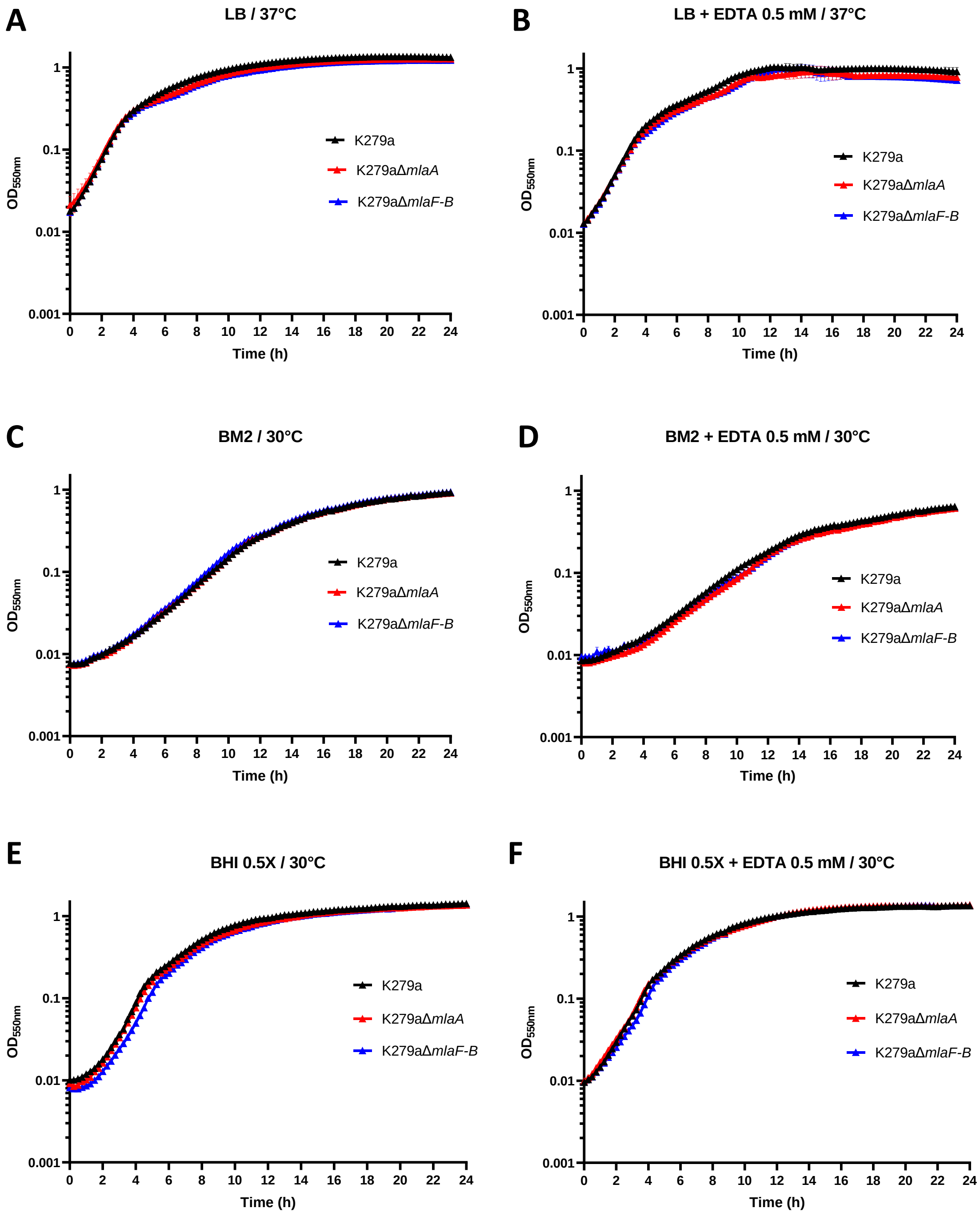

Figure S6. Growth curves of *S. maltophilia* K279a and the mutant strains K279a Δ*mlaA* and Δ*mlaF-B* under different growth conditions. Bacterial cells were grown in LB at 37°C **(A and B)**, modified BM2 at 30°C **(C and D)** or 0.5 × BHI at 30°C **(E and F)**, without and with 0.5 mM EDTA, and with continuous shaking. For all experiments, overnight cultures were diluted to an optical density at 550 nm (OD_{550nm}) of 0.01 to inoculate a 96-well microtiter plate (triplicate samples). The plates were incubated inside a Multiskan FC microplate photometer (Thermo Fisher Scientific) under constant circular shaking for 24 h, and the OD_{550nm} was measured every 15 min.

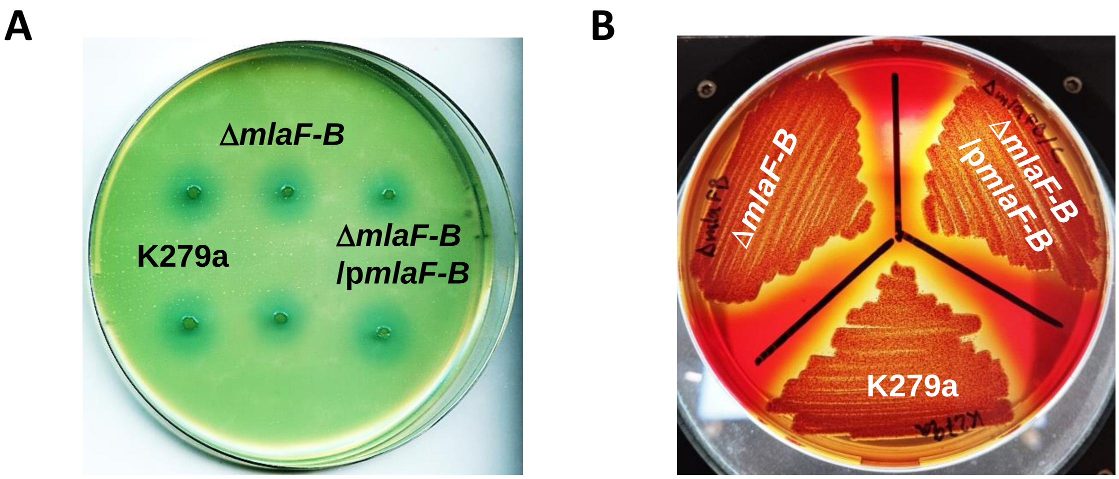

Figure S7. Different phenotypes of *S. maltophilia* K279a wild-type, its isogenic mutant strain Δ*mlaF-B* and the complemented strain Δ*mlaF-B*/p*mlaF-B*. **(A)** Colony DSF bioassay on NYG agar plates containing 80 µg/mL X-Glu and the reporter strain *Xanthomonas campestris pv. campestris* (*Xcc*) 8523 pL6engGUS. **(B)** Growth on 0.08% (wt/v) Congo red agar plates. After growth for 24 hours at 30°C, the bacterial biomass absorbed the Congo red dye and produced red colonies consistent with exopolysaccharide production. Complementation plasmid pBBR1MCS1-*mlaF-B* is indicated as p*mlaF-B*.

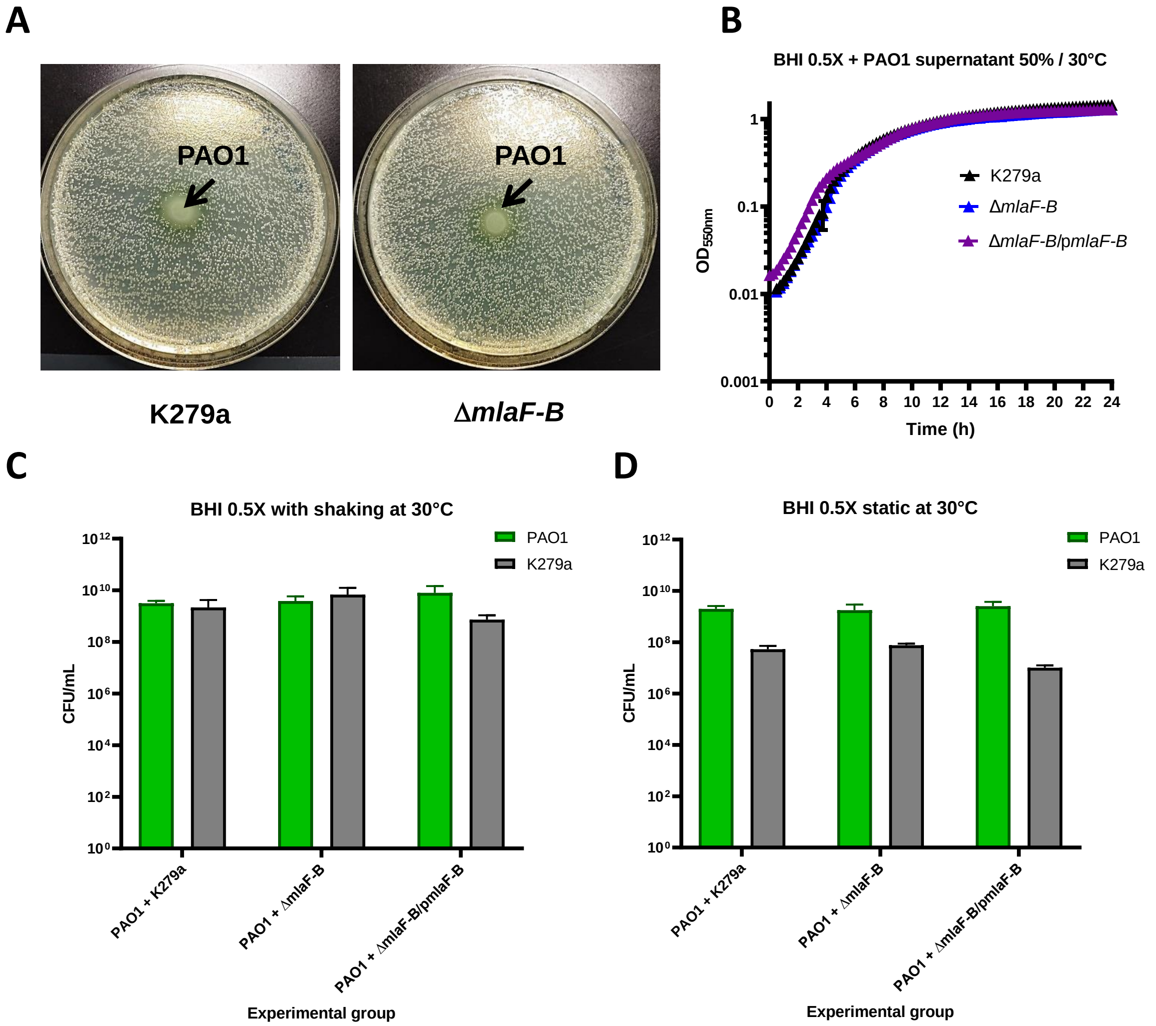

Figure S8. Competition between *P. aeruginosa* PAO1 and *S. maltophilia* K279a wild-type, the mutant (Δ*mlaF-B*) and complemented (Δ*mlaF-B*/p*mlaF-B*) strains under standard laboratory growth conditions. **(A)** Interspecies growth competition assay on LB agar plates. Macrocolonies of *P. aeruginosa* PAO1 grown on a lawn of *S. maltophilia* colonies. **(B)** Growth curves of *S. maltophilia* strains in 0.5 × BHI medium at 30°C and supplemented with 50% filtered supernatant of PAO1. **(C and D)** Quantification of the cell population of *S. maltophilia* and *P. aeruginosa* in mixed cultures with or without shaking. Samples were collected after 24 hours of incubation in 0.5 × BHI medium at 30°C, serial diluted in PBS, and then seeded onto LB agar plates with appropriate antibiotics, except for the complemented variant that was differentiated by colony morphology. Complementation plasmid pBBR1MCS1-*mlaF-B* is indicated as p*mlaF-B*.
